# The phenotypic impact of the male-specific region of chromosome-Y in inbred mating: the role of genetic variants and gene duplications in multiple inbred rat strains

**DOI:** 10.1186/s13293-016-0064-z

**Published:** 2016-02-03

**Authors:** Jeremy W. Prokop, Shirng-Wern Tsaih, Allison B. Faber, Shannon Boehme, Adam C. Underwood, Samuel Troyer, Lauren Playl, Amy Milsted, Monte E. Turner, Daniel Ely, Almir S. Martins, Marek Tutaj, Jozef Lazar, Melinda R. Dwinell, Howard J. Jacob

**Affiliations:** HudsonAlpha Institute for Biotechnology, 601 Genome Way, Huntsville, AL 35806 USA; Human and Molecular Genetics Center, Medical College of Wisconsin, Milwaukee, WI 53226 USA; Department of Physiology, Medical College of Wisconsin, Milwaukee, WI 53226 USA; Department of Biology, The University of Akron, Akron, OH 44325 USA; Department of Mathematics and Science, Walsh University, North Canton, OH 44720 USA; Núcleo de Fisiologia Geral e Genômica Funcional-ICB-Universidade Federal de Minas Gerais (UFMG), Belo Horizonte, Minas Gerais Brazil

**Keywords:** MSY, *Rattus norvegicus*, Inbred mating, *Sry*, *Med14y*, *Ube2q2y*, Gene duplications, Phenotypic variation

## Abstract

**Backgound:**

The male-specific region of chromosome-Y (MSY) contributes to phenotypes outside of testis development and has a high rate of evolution between mammalian species. With a lack of genomic crossover, MSY is one of the few genomic areas under similar variation and evolutionary selection in inbred and outbred animal populations, allowing for an assessment of evolutionary mechanisms to translate between the populations.

**Methods:**

Using next-generation sequencing, MSY consomic strains, molecular characterization, and large-scale phenotyping, we present here regions of MSY that contribute to inbred strain phenotypes.

**Results:**

We have shown that (1) MSY of rat has nine autosomal gene transposition events with strain-specific selection; (2) sequence variants in MSY occur with a 1.98-fold higher number of variants than other chromosomes in seven sequenced rat strains; (3) *Sry*, the most studied MSY gene, has undergone extensive gene duplications, driving ubiquitous expression not seen in human or mouse; (4) the expression profile of *Sry* in the rat is driven by the insertion of the *Sry2* copy into an intron of the ubiquitously expressed Kdm5d gene in antisense orientation, but due to several loss of function mutations in the *Sry2* protein, nuclear localization and transcriptional control are decreased; (5) expression of *Sry* copies other than *Sry2* in the rat overlaps with the expression profile for human *SRY*; (6) gene duplications and sequence variants (P76T) of *Sry* can be selected for phenotypes such as high blood pressure and androgen receptor signaling within inbred mating; and most importantly, (7) per chromosome size, MSY contributes to higher strain-specific phenotypic variation relative to all other chromosomes, with 53 phenotypes showing both a male to female and consomic cross significance.

**Conclusion:**

The data presented supports a high probability of MSY genetic variation altering a broad range of inbred rat phenotypes.

**Electronic supplementary material:**

The online version of this article (doi:10.1186/s13293-016-0064-z) contains supplementary material, which is available to authorized users.

## Background

Recent analysis of the male-specific region of chromosome-Y (MSY) has identified a core set of genes found throughout mammalian evolution [1]. While many of these genes contribute to classical sex determination and testis function in mammals, they additionally contribute to the maintenance of X-Y gene expression levels and numerous sex disparities in diseases [2, 3]. The association of human age-related loss of MSY with mortality age in males [4], in combination with a ubiquitous expression profile for several human MSY genes [3], signifies functional importance of genes residing on MSY to normal biological functions outside of sex determination. Disease associations for MSY include cardiovascular disorders, asthma, autoimmune disorders, birth defects, neurological/psychiatric disorders (including schizophrenia and Parkinson disease), and many cancers [3, 5]. Due to the lack of the MSY to undergo recombination, it is one of the largest linkage disequilibrium (LD) blocks within the human genome; therefore, identifying the causal variants from haplotypes is very difficult using human genomics alone, making it as difficult as autosomal variant analysis before the completion of the 1000 Genomes Project. With the complex genetics of the MSY, research into this field has also been slowed by the lack of animal congenic mapping and the lack of high-throughput characterizations in MSY animal consomic models.

However, the high mutation rate within MSY and an identical inheritance pathway between outbred and inbred animals could allow for comparative genomics in animal models to help identify MSY genes that are involved in phenotypes outside of sex determination, and therefore could be used to look for variants in human that contribute to disease. To date, no animal models with both genetics and high-throughput phenotyping have been developed to study these broad phenotypic contributions of MSY genetics.

**Fig. 1:**
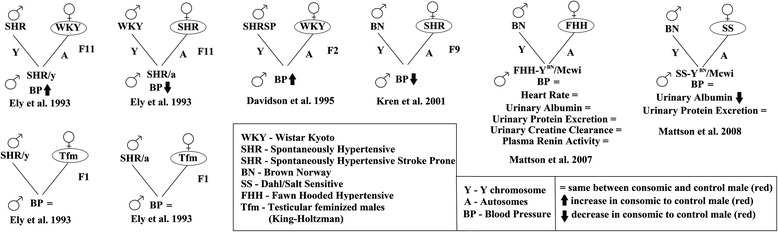
MSY consomic rats that have been generated. Crosses of male (♂) and female (♀) rat strains have generated six different MSY consomic strains. Abbreviations for rat strains are shown in the *first box*. Phenotyping of the animal was performed at the designated generation (F11, F2, F9) relative to a male from the strain designated in a circle. For two of the consomic strains, they were crossed with the Tfm rat, containing a loss of function androgen receptor mutation, to assess the segregation of blood pressure contributions due to MSY vs. MSY and hormone signaling

Sex differences in cardiovascular disease have been shown previously [6]. MSY haplotypes in human correlate with increased risk of coronary artery disease and blood pressure [7, 8]. The rat (*Rattus norvegicus*) has been a model organism for MSY contributions to blood pressure regulation for 25 years [9], but has lacked MSY sequence information until 2014, making it impossible to determine mechanisms of MSY phenotypic contributions. Consomic rats generated introgressing MSY between common strains of rats by our group and others for SHR to WKY [10], WKY to SHR [10], SHRSP to WKY [11], BN to SHR [12], BN to FHH [13], and BN to SS [14] have suggested strain-specific contributions of the rat MSY to blood pressure and kidney function (Fig. 1, strain abbreviations can be found in the figure). An interplay between MSY and androgen receptor signaling has been previously suggested for the SHR blood pressure regulation [10]. An F1 cross of the SHR/y (consomic of the SHR MSY onto WKY autosomes) to the testicular feminized rat (Tfm) repressed the role of MSY on blood pressure [10], but no understanding of the mechanisms behind this has yet been discovered.

Due to a high mutation rate of MSY, combined with a lack of chromosomal crossover, it is hypothesized that many inbred rat strains have had genetic variants on MSY selected upon for phenotypic traits such as blood pressure. These genetic variants of rat MSY may influence similar genes as those involved in human disease or MSY evolution throughout speciation, allowing researchers to narrow down the large human haplotype block to individual genes involved in these processes. As the rat commonly serves as a model for understanding sex differences through hormones, it is critical to define the genetics of strain-specific changes that may also serve to alter sex differences, resulting in a fuller understanding of both genetic and hormone contributions to sex differences in our model organisms. Therefore, we have undertaken an analysis of MSY genes in the most commonly researched strains of rat, which have previously had autosomal genomes sequenced [15]. The characterization of genes within rat MSY, variants within multiple inbred lines, molecular characterization of several protein-coding variants, and a large-scale analysis of MSY phenotyping for several strains will contribute to future development of animal models to study the role of MSY genes.

## Methods

### RNAseq analysis for *Sry* and other chromosome-Y genes

Analysis of RNAseq datasets for *Sry* was initially performed on publically available RNAseq datasets in NCBI for *R. norvegicus* differentiating *Sry2* from the other *Sry* copies*.* This was performed using the Sequence Read Archive (SRA) Nucleotide BLAST tool of NCBI optimized for megablast using Not_Sry2, Not_Sry2_2, Sry2, and Sry2_2 sequences of Additional file 1: Table S1. All positive reads were 100 % homologous with an *E*-value <9e−12. Positive reads for each RNAseq experiment were normalized with total number of reads in the SRA file and shown as reads per million (RPM). BLAST analysis was also performed using the Rat BodyMap datasets of the Fischer 344 rat RNAseq for 11 tissues of four ages in both males and females [16]. Additional sequences for *Sry* copy specificity (Additional file 1: Table S1) were used in the analysis to determine relative expression of each copy. The single-nucleotide variants (SNVs) in these sequences were placed in the middle or on the ends of the sequence such that reads with 100 % homology and an *E*-value <9e−12 all contained the SNV. One female genome (LE- ERR224452) was used as a negative control for all MSY variant sites.

Utilizing the SNVs in the male to female genome analyses below, SNVs were identified that are within 20 bases of each other for the various male-specific SNVs. These sites then had a BLAST analysis done against HTGS files of the rat to confirm they are present on chromosome-Y BAC sequences. Then using these sequences with the SNVs (Additional file 1: Table S1), the 160 male RNAseq files of the Rat BodyMap were analyzed for tissue expression using BLAST with the above parameters.

### Analysis of whole-genome sequencing data for chromosome-Y SNVs and indels

The DNA of seven male rat genomes (ACI, FHH, FHL, SBH, SBN, SR, and SS) was sequenced with the Illumina HiSeq2000 platform (Illumina, Inc., San Diego, CA) at the Medical College of Wisconsin. The Rnor_6.0 rat assembly containing the SHR/Akr chromosome-Y was obtained and indexed with BWA (version 0.7.7). Paired-end reads of each male rat genome were aligned to Rnor_6.0 reference sequences with BWA-MEM (version 0.7.7). Read pairing information and flags in the alignment were further cleaned up with fixmate in SAMtools (version 1.1). Duplicates were marked with Picard (version 1.108) prior to variant detection. Variants were called using the Genome Analysis Toolkit (GATK) UnifiedGenotyper (version 3.2.2) with the parameter setting suggested by the best practice (https://www.broadinstitute.org/gatk/guide/best-practices?bpm=DNAseq).

### DNA analysis for chromosome-Y genes in multiple rat strains

Analysis of *Sry* copies in genomic DNA reads of eight male rat strains (SHR- ERR224462, WKY- ERR224468, FHH- ERX199109, FHL- ERX199110, ACI- ERX199106, SR- ERX199124, SS- ERX199126, Fischer 344- ERR224448) was performed by BLAST analysis of SRA files using *Sry*-specific SNVs to differentiate the multiple copies (Additional file 1: Table S1). One female genome (LE- ERR224452) was used as a negative control.

Whole-genome sequence reads from 20 inbred rat strains (BBDP, LE/Stm, MHS, MNS, WAG, ACI, F344/NCrl, FHH, FHL, LEW, LEW/NClrBR, LH, LL, LN, SBH, SBN, SHRSP/Gla, SR/Jr, SS/Jr, SS_JrHsdMcwi) were aligned to the rat reference genome Rnor_5.0 (lacking MSY) using BWA, and SNVs were identified in all samples using GATK. The ratio of alternative to reference reads in each strain was calculated from the GATK variant calls to determine zygosity at each locus. With a compiled list of SNVs, we also determined if the genomic DNA sequenced for each of these 20 strains was male (and male contaminated female) or female with BLAST analysis of the reads using the *Sry1* coding sequence. In order to identify male-specific SNVs in the whole genome, those genomes that were female were then set to allele frequency of 0 % for SNVs while the male genomes were set to anything between 0.1 and 99.9 %. Additionally, we choose to identify strain-specific SNVs in the males again using 0 % allele frequency for the female genomes, setting one male allele frequency at a time to 0 %, and setting all other male allele frequency to 0.1–99.9 %. For each variant, the average allele frequency was calculated for all male rat strains for each SNV and then averaged over the multiple SNVs located within a single gene, calculating the standard error of the mean (SEM) of the later.

### *Sry* expression using real-time PCR and fragment analysis

Male 15–20-week-old rats were anesthetized by intramuscular injection of 2.5 % sodium pentothal at a concentration of 2 μL per gram body weight and then terminated by decapitation. Tissues were isolated and stored at −80 °C until use. RNA was isolated using RNA STAT 60 (Tel-Test Inc, Friendswood, TX) and precipitated with isopropanol. The removal of residual DNA from RNA samples by DNase was carried out using TURBO DNA-free DNase enzyme (Ambion, Waltham, MA). RNA concentration and quality was determined with a Nanodrop ND-1000 Spectrophotometer. Reverse transcription of RNA was performed with ArrayScript Reverse Transcriptase (Ambion) and RtallS-A primer (5′- GGACAGTAAGTAGGTTAGCT-3′) that is *Sry* and strand specific. Real-time PCR was performed with SYBR Green (Applied Biosystems, Waltham, MA) using *Sry* L (5′-GCG CCC CAT GAA TGC AT-3′) and *Sry* R (5′-TGG GAT TCT GTT GAG CCA ACT-3′) primer set on an ABI Prism 7700 Real-Time PCR System. ΔCT values were analyzed via two-way ANOVA, tissue by strain with age and rat number as random effects, followed by Tukey post hoc test. Differences were considered significant at *P* < 0.05. Fragment analysis was performed as previously described [17]. In short, using the cDNA of above following by PCR reactions with GoTaq Flexi DNA Polymerase (Promega, Fitchburg, WI) with 5'S2L (5′-CCA TCT CTG ACT TCC TGG TTG-3′) and RtallS-B (5′-AGT AGG TTA GCT GCT GCT AG-3′) primers. The amplicon was then labeled with three separate PCR reactions to differentiate the copies using NED-*P1mod(5′-GAA TGC ATT TAT GGT GTG GTC CCG-3′) with S1502G1rev(5′-TAG TGG AAC TGG TGC TGC TG-3′), dCAP-*Sry*1 *Hind*III(5′-AGA ATT CAG AGA TCA GCA AGC T-3′) with S1502G1rev*-VIC(5′-TAG TGG AAC TGG TGC TGC TG-3′), and 5'S2L with M1*-FAM(5′-TTT GTT GAG GCA ACT TCA CGC TGC-3′). The dCAP reaction was digested with *Hinf*I restriction enzyme. Samples were run on an Applied Biosystems 3130*xl* Genetic Analyzer using 5.5 % *v*/*v* GeneScan LIZ 600 sizing standard (Applied Biosystems). Data was interpreted with GeneMapper version 4.0.

### SRY2 luciferase and cellular localization studies

The Sry responsive luciferase reporter, pGL3/AR600, was produced by amplifying and inserting 590 bases of 5′ UTR from rat androgen receptor, isolated from a single ♂SHR/y rat with primers 5′-GTA CCA TGG TTT AGC TTG TCT CTA GCT TCC ACC-3′ and 5′- CAC CCG GGT AAC TCC CTT TGG CTG A-3′. Amplicons were cleaved using endonucleases *Nco*I and *Sma*I, and the resulting restriction fragments were then gel extracted and inserted into pGL3 vector (Promega) opened with the same enzymes. Assembly of all native pEF1/*Sry*1, 2, and 3 effector constructs and truncated or site specifically mutated effector constructs was generated.

Chinese hamster ovary (CHO) cells were cultured at 37 °C in HAM’s F12K medium (Sigma-Aldrich, St. Louis, MO) supplemented with 10 mM HEPES and 10 % FBS (Atlanta Biologicals) in a humidified atmosphere with 5 % CO_2_. Prior to cotransfection, cells were seeded to 24-well cassettes (6.6 × 10^3^ cells/cm^2^) and incubated for 16 h. Each well was transiently cotransfected with 50 ng effector plasmid, 500 ng firefly luciferase reporter (pGL/AR600), and 500 pg of control construct, phRL-null *Renilla* (Promega), using SuperFect transfection reagent (Qiagen, Venlo, Netherlands) following the manufacture’s protocol. After 24 h incubation, CHO cells were processed for luciferase activity using the reagents and protocol provided in the Dual-Luciferase® Reporter (DRL™) Assay System (Promega). Luciferase to Renilla ratios were obtained from measurements collected on a Turner Biosystems 20/20^*n*^ luminometer and were used to calculate pGL3/AR600 reporter activity of each *Sry* effector construct relative to reporter activity obtained from CHO transfected with an pEF1/Myc C vector containing no insert. Data reported represent means ± SEM of three trials conducted in triplicate with each *Sry* effector construct. Statistical analysis was performed by using one-way ANOVA and a post hoc Student-Newman-Kuels test and Student’s *t* test where applicable. Analyses were run on SigmaStat software (Jandel Scientific, San Rafael, CA) with significance assumed at *P* < 0.05.

Various Sry constructs were created from Sry1 and Sry2 pEF1 protein expression vectors to compare regions that differ between Sry1, Sry2, and Sry3 proteins. These constructs were Sry1(HMGbox)—Sry1 with only the HMGbox, Sry1(delPolyQ)—Sry1 glutamine-rich region converted to that of Sry2, Sry2(-QR)—removal of the entire glutamine-rich region, Sry1(20-22AAA)—alanine mutations to the N-terminal nuclear localization motif of Sry1, Sry1(78-79AA)—alanine mutations to the C-terminal nuclear localization motif of Sry1, Sry1(NoNLS)—alanine mutations to both the N- and C-terminal nuclear localization motifs of Sry1, Sry1(H21R)—histidine to arginine mutation corresponding to the site seen in Sry2, and Sry2(H21R)—histidine to arginine mutation corresponding to the site seen in Sry1. CHO cells grown to approximately 1 × 105 cells/cm^2^ were transfected with 7.5 μg of each respective plasmid DNA using ExGen500 transfection reagent (Fermentas), incubated for 24 h, trypsinized/pelleted, and cytoplasmic/nuclear extracts were prepared using ProteoJET Cytoplasmic and Nuclear Protein Extraction Kit (Fermentas). Cytoplasmic and nuclear protein extracts (20 μg) were separated on 13.5 % polyacrylamide gels. Proteins were transferred to PVDF membranes that were blocked for 1 h at room temperature in PBS containing 5 % nonfat dry milk and 0.1 % Tween-20. SRY proteins where detected using a goat anti-mouse SRY (Santa Cruz Biotechnology, Inc., Dallas, TX) or a goat anti-Myc epitope (Bethyl Laboratories, Montgomery, TX) antibodies, diluted in a blocking solution at 1:300 and 1:1000, respectively. After a 1-h incubation at 22 °C, blots were washed in PBS, following a 1-h incubation with a donkey anti-goat HRP conjugate (Bethyl Laboratories) diluted to 1:3000 in blocking solution. Bands were detected using SuperSignal West Pico Chemiluminescent substrate (Thermo Fisher Scientific Inc.) and visualized with a Kodak 2200 Gel Logic Imaging system. All assays included a control lane containing cell extracts obtained from cells transfected with an expression vector containing no insert.

### Protein modeling

Models for the nonHMGSry protein were generated using the ab initio modeling server Quark [18]. Each of the top five models were run for 10 ns of molecular dynamic simulations using YASARA with the Amber03 force field [19], 0.997 g/mL water, p*K*a of 7.4, and mass fraction of 0.9 %. The *Z*-score, wrong isomers, and *cis*-peptide bonds were calculated using the YASARA2 force field. Combining these calculations with the analysis of movement throughout the molecular dynamic simulations, the models were ranked to determine the most likely structure. The nonHMGSry protein sequence was also analyzed for functional motifs using ELM [20].

### SRY-AR synergistic regulation assay

Luciferase assays on the *Sry1* and *AR600* promoters were performed and analyzed as previously published [21]. The SRY/AR synergistic regulation experiments were performed using charcoal stripped fetal bovine serum (Innovative Research, Novi, MI) with or without the addition of 100 nM testosterone (Sigma-Aldrich). The hSRY P131T corresponds to the variation seen in SRY1 (P76) to SRY3 (T76) [22]. Androgen receptor (AR) expression vector was purchased from ATCC (vector #80005, hARa [CL7a-AR 160–910]). For each transfection, 100 ng of expression vector was used per well with single protein expressions using 50 ng of vector (hSRY, AR, or P73T) with 50 ng of empty vector and double transfections (hSRY/P131T with AR) using 50 ng of each. All constructs were sequence confirmed on an ABI 3130xl using BigDye Terminator v3.1.

### Sry3 electroporation and blood pressure studies

A total of ten animals (normotensive WKY rats) were used for three experimental groups. There were three empty vector animals and seven Sry3-treated animals. All of the empty vector animals and four of the Sry3 animals received drug treatment with olmesartan medoxomil. Animals received a standard 12-h light/dark cycle and were given standard rat chow (22.5 % protein, 52 % carbohydrate, and 6 % fat by weight, Prolab 3000, Agway, Syracuse, NY) and water ad libitum. Rats were individually housed in polycarbonate cages (48 cm × 27 cm × 20 cm) with heat‐treated bedding (Sani Chips, R.J.Murphy, Rochelle Park, NJ). Cage changes were performed once a week, scheduled so as not to interfere with blood or urine sampling.

Animals were implanted with an aorta telemetry device (model-PAC40; Data Sciences International, St. Paul, MN), and baseline measurements of systolic pressure, diastolic pressure, heart rate, and activity were collected (RPC-1 and Dataquest A.R.T., Data Sciences International). All animals were monitored for telemetry measurements throughout the study, at 30‐min intervals, except for the 24‐h periods that animals spent in metabolic cages. Animals were allowed to recover from telemetry surgery for 1 week before a baseline metabolic cage study was done.

All animals were injected and electroporated with PEF1(−) or PEF1/Sry3 (25 μg) into the left kidney as previously performed [23], which represents day 1 of the study. On day 6, the first 24‐hour urine was taken followed by the first plasma sample on day 8. On day 14, animals were given sham- or olmesartan-treated drinking water. The drug was given for 1 week total, and on day 17, a second 24‐h urine was taken followed by a second plasma on day 19. Drug treatment was then stopped and time was given to allow the drug to leave the system. On day 25, a final 24‐h urine was collected followed by a final plasma sample on day 27. All the above animal experiments were approved by the University of Akron IACUC.

### Phenotype analysis of BNxFHH and BNxSS consomic panels

The chromosome-Y consomic rats were generated as part of the PhysGen Program for Genomic Applications (http://pga.mcw.edu). Detailed phenotyping protocols are posted on the website. Briefly, six phenotyping protocols (Lung, Respiratory, Cardiac, Renal, Vascular, Biochemistry) were run in parallel using ten male and ten female rats per protocol. Rats were studied between 6 and 10 weeks of age and under control conditions or under diet or environmental stress. The consomic rats were studied using the same protocols and quality controls as previously described [24]. Phenotypes listed to significantly differ were determined using adjusted *P* values <0.05 in the consomic strain (for example SS-Y^BN^/Mcwi) compared to the parental strain (for example the SS). The adjusted *P* values were calculated by the Mann-Whitney test followed by a Bonferroni adjustment for multiple tests. All animal experiments were approved by the Medical College of Wisconsin IACUC.

## Results

### Mammalian conserved protein-coding genes in rat

**Fig. 2:**
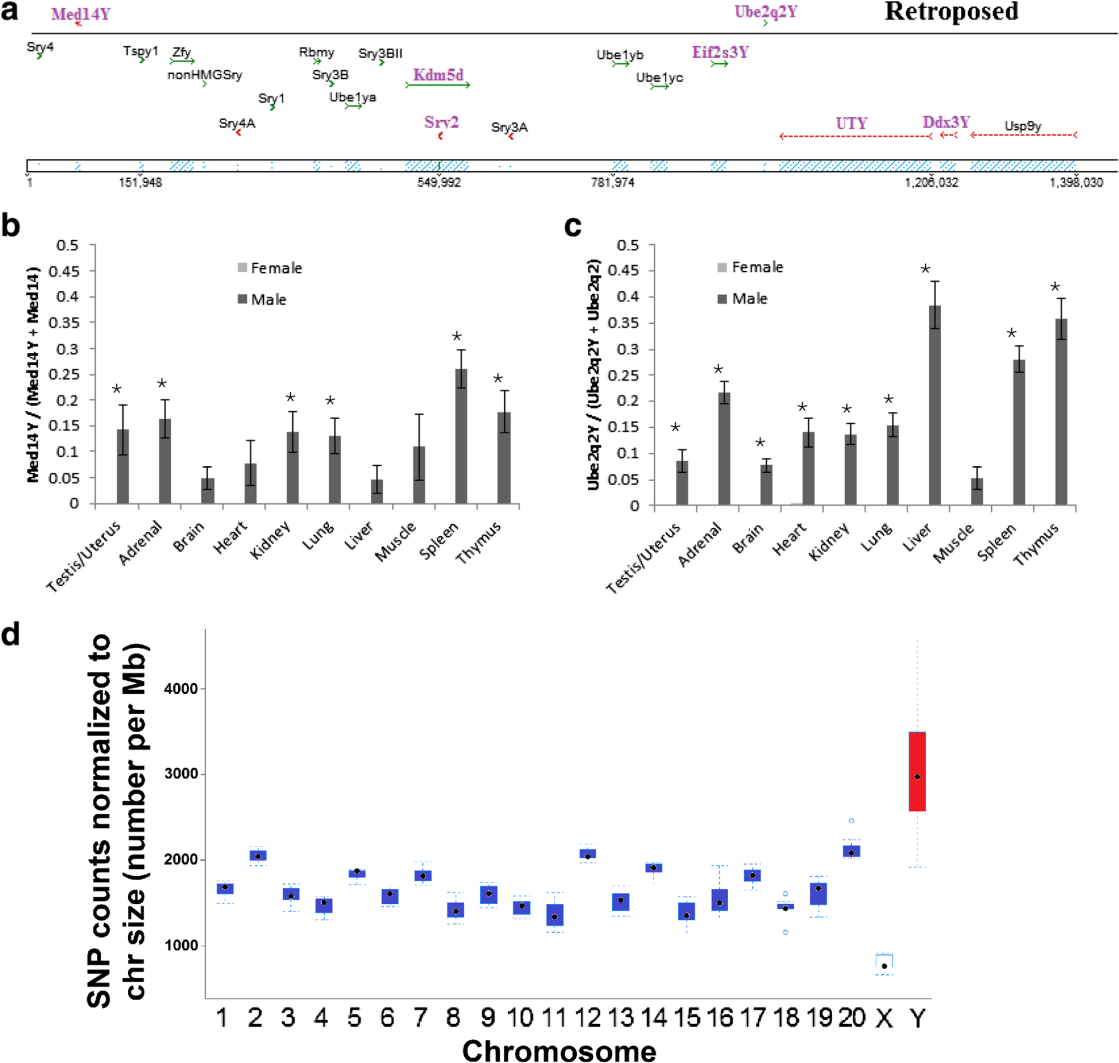
Rat MSY genes. **a** Alignment of the various genes described in this paper onto the RNor 6.0 MSY sequence of *Rattus norvegicus*. Retroposed genes that align onto the sequence are shown on the *top*. Ubiquitously expressed genes in the Rat BodyMap dataset are colored in *purple* while tissue-specific genes are in *black*. **b**, **c** Expression profiling for the F344 Rat BodyMap dataset for *Med14Y* (**b**) and *Ube2q2Y* (**c**) in multiple tissues of male and female animals. Expression is shown as the number of reads for MSY copy (*Med14y*/*Ube2q2y*) divided by X (*Med14y*) or chromosome 8 (*Ube2q2*) and Y copies, in such that values of 0.5 are a 50/50 ratio of transcripts from the X/8 and MSYs and a value of 0 is expression from only the X/8-chromosome. *Error bars* represent the error of four independent RNAseq datasets and *asterisk* represents a *P* < 0.05 between male and female rats. **d** Distribution of SNPs detected in seven male rat strains on the genome. SNP counts on the *y*-axis of box-and-whisker plot are normalized to the chromosome size in Mb. MSY (red) is shown to have highly elevated number of mutations compared to all other chromosomes

Of mammalian conserved MSY genes, nine (*Eif2s3y*, *Zfy*, *Usp9y*, *Ddx3y*, *Uty*, *Ube1y*, *Kdm5d*, *Rbmy*, *Sry*) are confirmed in rat [2] and thus would be primary MSY genes of interest in translatable human disease. All nine conserved genes are expressed in rat testis, with five genes (*Eif2s3y*, *Ddx3y*, *Uty*, *Kdm5d*, and *Sry)* expressed in all ten male tissues of the Rat BodyMap project (Table 1). *Sry* is well known to have undergone gene duplication, with variants defining 11 genes: *Sry1*, *Sry2*, *Sry3*, *Sry3A*, *Sry3B*, *Sry3BI*, *Sry3BII*, *Sry3C*, *Sry4*, *Sry4A*, and *nonHMGSry* [21]. Most of these SNPs for individual *Sry* genes could be mapped onto the rat MSY sequence relative to other human shared genes (Fig. 2a).

**Table 1:** Conserved Y chromosome genes found in rat

	Accession number	Y chromosome SHR BAC	Primary tissue expression in rat
EIF2S3Y	GATN01000003.1	AC242953.5	Ubiquitous
ZFY	GATN01000010.1	AC239865.4	Testis
USP9Y	GATN01000001.1	AC242055.4	Testis
DDX3Y	GATN01000002.1	AC242055.4	Ubiquitous
UTY	GATN01000009.1	AC241873.4	Ubiquitous
UBE1Y	EF690356.2	AC242953.5	Testis
KDM5D	GATN01000004.1	AC242859.2	Ubiquitous
RBMY	GATN01000006.1	AC239817.2	Testis
SRY	Multiple	AC239701.6	Ubiquitous

### Autosomal gene duplications onto the rat MSY

While identifying *Sry* copies in the SHR MSY sequence, we found transposable elements were likely causal for duplication of *Sry* genes and also identified two non-MSY genes, *Med14Y* and *Limd2Y*, that have been duplicated from the X chromosome and autosomes onto the rat MSY [21]. However, the confirmation of these and other duplicated genes onto rat MSY is difficult due to repetitive sequence. To confirm duplicated genes, we developed a novel sequence analysis, comparing variants of genes identified in males but not females, using whole-genome sequencing.

The absolute sex for all reads of 23 sequenced rat genomes was determined, identifying *Sry* sequence reads in 18 genomes; thus, only 5 genomes are 100 % female DNA (Additional file 1: Table S2). This approach allows for *Sry*-contaminated female sequences to be treated as males, thus treating SNPs of duplicated genes of males that could also be included with equal probability as *Sry* sequence. Following alignment of reads to the Rnor 5.0 genome (lacks MSY sequence), all single-nucleotide variants (SNVs) found in protein-coding genes of male genomes and not female genomes identified a subset of genes potentially inserted onto MSY (Table 2). Nine genes are found to have specific SNVs in all 18 male rat genomes with an average allele frequency of 30.7 % over 2790 SNVs, close to the 33 % expected frequency for a variant found in an autosomal gene that has been duplicated onto the MSY. The duplicated *Limd2* was confirmed by this analysis for all strains; however, *Med14* was not. Removing individual male genomes from the analysis, we were able to identify strain-specific variants in male duplicated genes, such as the lack of 33 *Med14* SNVs in the FHL rat strain, suggesting this gene is not present in the FHL MSY.

**Table 2:** Autosomal gene duplication to MSY

Chromosome	Gene	SNVs	Average allele frequency	Strain w/o SNVs	Strain-specific SNVs	MSY SHR/Akr BAC	MSY gene	Duplication method	Tissue expression
chr2	*Ect2*	25	23.9 ± 2.3	–	SBH(2), SR(2), SS(1)	AC241473.5	*Ect2Y_ps*	Retroposed	None
chr8	*Ube2q2*	16	20.6 ± 2.1	–	LL(2)	AC241873.4	*Ube2q2Y*	Retroposed	Ubiquitous
chr10	*Havcr2*	33	30.8 ± 2.3	–	–	AC241887.5	*Havcr2Y_ps*	Retroposed	None
chr10	*Limd2*	40	45.2 ± 2.9	–	LH(1), SBN(1)	AC241808.7	*Limd2Y_ps*	Retroposed	None
chr12	*RGD1560580*	4	24.9 ± 4.5	–	–	AC241548.6	*Ssty2*	MSY duplication	None
chr13	*Xpr1*	24	28.9 ± 2.1	–	F344(2), SBN(1)	AC246656.3	*Xpr1_Y1/2/3/4*	Transposed	None
chr13	*Prrc2c*	2	19.6 ± 1.8	–	–	AC241950.2	*Prrc2cY_ps*	Retroposed	None
chr14	*Vom2r67*	5	24.4 ± 2.7	–	SBH(2), SR(1)	AC242505.8	*Vom2rY_ps*	Retroposed	None
chr16	*Lsm1*	5	24.9 ± 2.2	–	–	AC243283.4	*Lsm1Y_ps*	Partial transposed	None
chrX	*Med14*	33	31.2 ± 3.3	FHL	FHH(1)	AC239701.6	*Med14y*	Retroposed	Ubiquitous

Clustered male-specific SNVs from the analysis above (two or more SNVs located within 20 bases), allowing for single-read detection of multiple variants found throughout male rat strains, were used to screen MSY bacterial artificial chromosomes (BACs) and also the Rat BodyMap datasets. Each of the specific SNV sites was detected in SHR MSY sequencing BACs (Table 1), confirming with a secondary sequencing approach the existence of the duplicated genes into the MSY. This identification on MSY BACs also allowed for a prediction of the mechanism of duplication. Seven genes were identified as retroposed (i.e., spliced genes reinserted onto MSY), one as a MSY gene that duplicated to an autosome (*RGD1560580*) and two as transposed genes (i.e., contains normal introns of gene). Using the Rat BodyMap dataset in combination with the male-specific SNVs, expression was detected for only two retroposed genes, *Med14Y* (Fig. 2b) and *Ube2q2Y* (GenBank KM610331, Fig. 2c), in male and not female RNAseq datasets.

*Med14Y* (Fig. 2b) and *Ube2q2Y* (Fig. 2c) are expressed in all male but not female tissues of the Rat BodyMap dataset, with their MSY location shown on Fig. 2a. In comparison to *Med14Y* and *Ube2q2Y*, *Limd2Y* has only three detectable reads out of the 13.3 billion analyzed and also contains one mutation resulting in the deletion of a Zn coordination site required for structural folding [25] and additional nonsense mutations (Additional file 1: Figure S1). The combination of a lack of transcription and mutations that would inhibit protein function suggests that *Limd2Y* has become a pseudogene (*Limd2Y-ps*) on rat MSY in all sequenced male strains. Initial analysis of our rat variant visualizer tool, based on Rnor 3.4 genome alignment lacking MSY sequence, on the rat genome database (http://rgd.mcw.edu/rgdweb/front/config.html) for all strains showed that these variants for all autosomal genes inserted into the MSY were present at around 1/3 allele frequency in the database. This suggests a possibility of researchers to misidentify MSY gene variants as heterozygous variants in autosomal genes.

To update this information and add ChY gene variants between strains, a new strain genome comparison tool was created. We sequenced male genomes for seven strains (ACI, FHH, FHL, SBH, SBN, SR, SS) and aligned them onto the Rnor 6.0 assembly that includes newly sequenced MSY. The number of single-nucleotide variants (SNVs) was calculated for each strain and was made relative to total chromosome size (Additional file 1: Table S3). The analysis shows a 1.98 ± 0.06-fold elevation of SNVs on MSY relative to all other chromosomes (Fig. 2d), a value that is similar with previous rodent MSY sequencing work [26] confirming MSY wide rate in nearly every analyzed rat strain. To resolve this issue with variant analysis, we have developed and released a new Variant Visualizer from the rat genome database (http://rgd.mcw.edu/rgdweb/front/config.html?mapKey=360) for variants called from Rnor 6.0 alignment for seven different rat strains.

### *Sry* duplicated genes in commonly used rat strains

*Sry* is expressed in numerous human tissues [3]. An overlap of many tissues, such as kidney, is seen for the expression of *Sry* in primates and rat that are not found in the mouse [27]. This expression is novel to *Sry* as it is not seen for *Sox3*, the ChrX homolog of *Sry* [27]. However, the expression profile of rat *Sry* is ubiquitous while in human it is not [3]. Unlike the human genome, the rat genome contains 11 functionally distinct copies of *Sry* (*http://bmcgenomics.biomedcentral.com/articles/10.1186/1471-2164-14-792*) likely amplified through gene conversion at repetitive elements [21]. The existence of these 11 copies has only been performed in two strains to date (SHR and WKY), and an expression profile for each copy has never been performed in detail before. Thus, the overlapping function between *Sry* in rat and human would be better understood by a copy-specific expression analysis in multiple laboratory strains of rats.

**Fig. 3:**
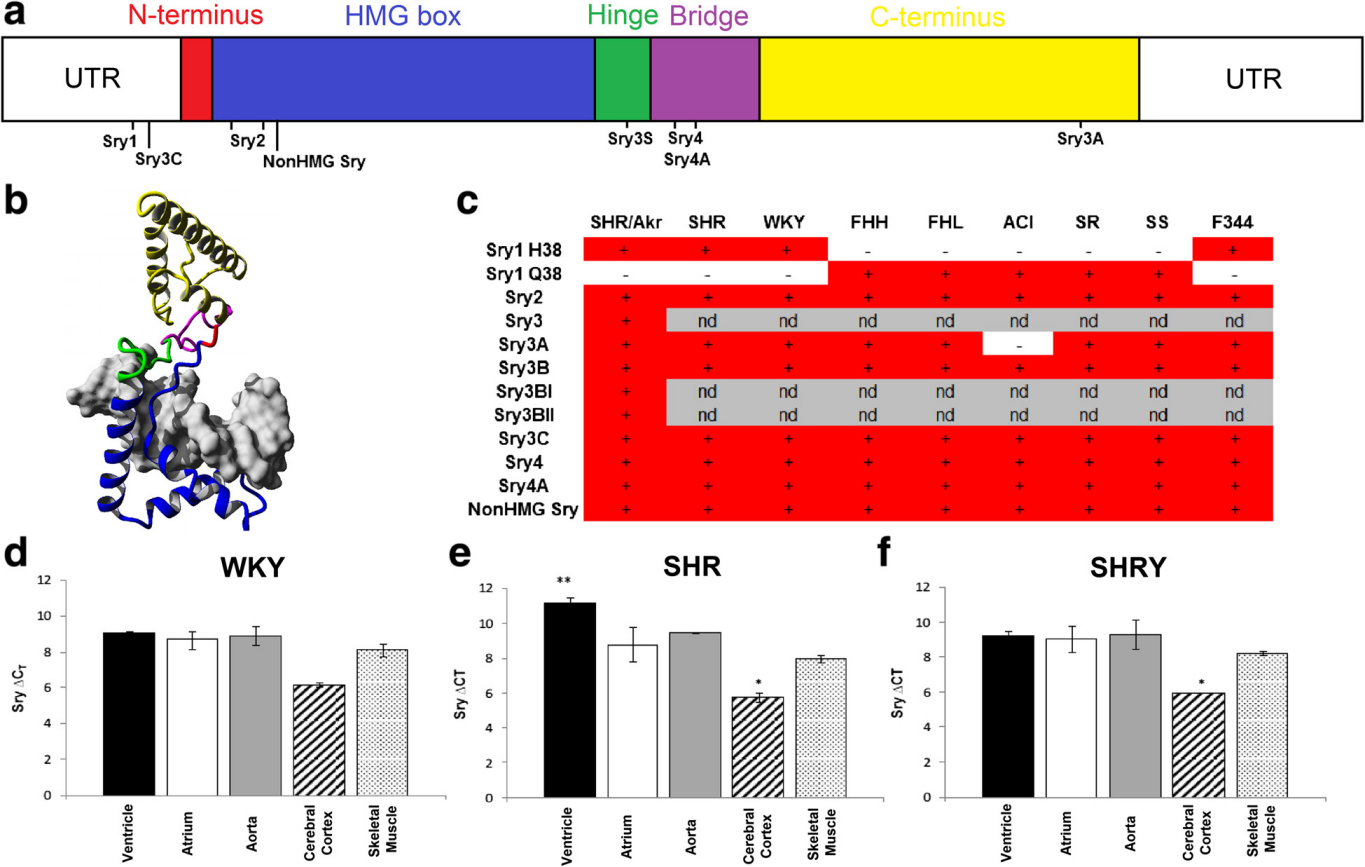
Characterization of the multiple *Sry* copies in commonly used rat strains. **a** Schematic breakdown of the domains found in rat SRY showing the N-terminus (*red*), HMG box (*blue*), hinge (*green*), bridge (*magenta*), and C-terminal (*yellow*) domains of the protein. Locations of SNVs that allow for the identification of individual copies based on the known SHR sequence are shown *below the schematic*. **b** Protein model of the full rat Sry1 protein bound to DNA (*gray*). Color scheme from **a** for the various domains are shown on the model. **c** Detection of the SNVs for the multiple copies of *Sry* from the genomic sequence reads for multiple male rat strains (SHR, WKY, FHH, FHL, ACI, SR, SS, and F334). The *plus sign* represents multiple reads with 100 % homology (*red boxes*), the *minus sign* represents those with no positive SNVs detected (*no color*), and *nd* are copies of *Sry* that do not contain SNVs allowing for identification using short reads. **d**–**f** Real-time PCR of Sry from five tissues for WKY (**d**), SHR (**e**), or the SHR/y consomic (**f**)

The advancing technology of next-generation sequencing allows for the identification of multiple *Sry* genes at sequence resolution in both genome and transcriptome, further enhancing our capabilities in segregating multiple *Sry* copies. Utilizing SNVs (Fig. 3a) unique to specific copies of rat *Sry* (protein shown in Fig. 3b), 8 of 11 *Sry* copies are confirmed in SHR, WKY, FHH, FHL, SR, SS, and F344 inbred rat strains (Fig. 3c). The remaining *Sry3* copy is unable to be differentiated (nd, not determined) from *Sry3BI/3BII* due to short reads used in next-generation sequencing. Our initial work using real-time PCR (Fig. 3d) confirms *Sry* multiple tissue expression in the rat; however, now that we have established the existence of at least 8 Sry copies in the majority of commonly used rat strains, a more detailed analysis of Sry copies expression is needed.

**Fig. 4:**
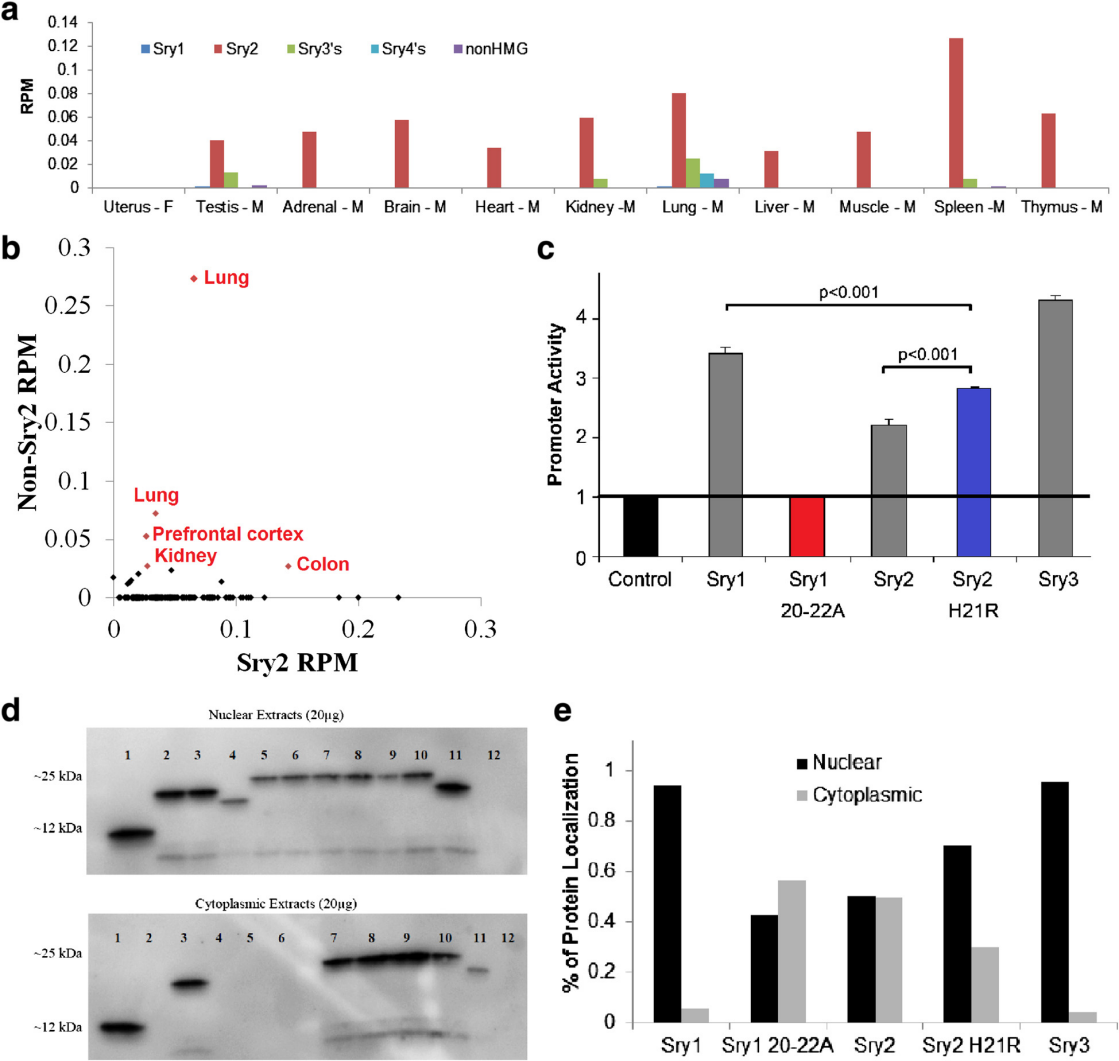
Expression and function of *Sry* copies. **a** Analysis of *Sry* transcripts (as reads per million sequence reads that contain identifying SNP) from the Rat BodyMap datasets for multiple tissues of males and a control female uterus tissue. Colors correspond to the Sry identifying SNP; *Sry1* = *blue*, *Sry2* = *red*, *Sry3’s* = *green*, *Sry4’s* = *cyan*, *nonHMGSry* = *magenta*. **b** Analysis of publically available rat RNAseq datasets (excluding the Rat BodyMap datasets) for the SNVs of *Sry2* (*x*-axis) vs. the other non-*Sry2* copies (*y*-axis). Tissues with the highest expression of non-*Sry2* copies are labeled in *red*. **c** Differences in transcriptional regulation by the SRY2 protein. Alanine mutations to nuclear localization site of Sry1 (Sry1 20-22A) results in a loss of promoter regulation (*red*). Mutating amino acid 21 from His (found in Sry2) to an Arg (found in Sry1 and Sry3) in the SRY2 protein results in a significant elevation of promoter activity (*blue*) relative to Sry2. **d**, **e** Nuclear localization of Sry constructs shown as Western blot for multiple Sry constructs (**d**) or quantification of western blot for several constructs (**e**). For panel **d**, *Lane 1* = Sry1(HMGbox), *2* = Sry1(delPolyQ), *3* = Sry2, *4* = Sry2(−QR), *5* = Sry1, *6* = Sry3, *7* = Sry1(20-22AAA), *8* = Sry1(78-79AA), *9* = Sry1(NoNLS), *10* = Sry1(R21H), *11* = Sry2(H21R), *12* = negative control (empty vector)

A fragment analysis protocol was initially developed to identify copy expression specificity, particularly for the *Sry2* gene. Using this approach, *Sry2* is observed to have the highest expression of *Sry* copies in most tissues [17]; however, this approach is limited to the analysis of only a few Sry copies that have variants altering restriction sites. The Rat BodyMap dataset allows, for the first time, the ability to assess expression of each *Sry* copy in multiple tissues (Fig. 4a). In agreement with our SHR and WKY fragment analysis, *Sry2* is the predominantly expressed copy throughout all tissues. To assess *Sry* expression in other rat strains, we utilized publically available RNAseq datasets in NCBI. *Sry* transcripts are detected in 197 separate rat RNAseq datasets; however, only 15 of these datasets contain transcripts that are from non-*Sry2* copies (Fig. 4b). This *Sry2* copy, found so ubiquitously transcribed, is located within an intron, in the antisense orientation, of the ubiquitously expressed *Kdm5d* gene (Fig. 2a) suggesting the location of the gene insertion has possibly driven elevated expression. Likely to compensate for this elevated expression, SRY2 rapidly accumulated an amino acid mutation (H21R) within the N-terminal nuclear localization site (nNLS) that alters the ability of SRY2 to activate transcription (Fig. 4c) and localize the protein within the nucleus (Fig. 4d, e). Additionally, we have previously shown that SRY2 variants located in the glutamine-rich region (deletion of 13 amino acids corresponding to changes in Sry2 location in SDS-PAGE of Fig. 4d) decrease transcriptional control [21]; however, these mutations and the complete removal of the glutamine-rich region (Fig. 4d, lane 4) do not alter nuclear localization (Fig. 4d), only transcriptional control.

Of the tissues analyzed from the Rat BodyMap and publically available RNAseq datasets, the testis, kidney, lung, spleen, brain, and colon show expression of non-*Sry2* copies. Interestingly, the list of rat tissues expressing non-*Sry2* genes (all Sry genes excluding *Sry2*) overlaps nearly perfectly with our previous analysis of the human protein atlas expression profile for human SRY [3]. The lung contains a vast array of *Sry* copies (Fig. 4a) including the highly conserved *nonHMGSry* (GenBank KC215141.1) that contains a frame shift mutation directly before the high-mobility group (HMG) domain, but still codes for an open reading frame. This is the first time transcripts from *nonHMGSry* have been identified. Protein modeling, molecular dynamic simulations, and functional motif analysis of this novel protein elucidate regions of structural order, possible nuclear localization motif, 14-3-3 binding motif, and a degradation box suggesting functional impact for transcripts from this rat conserved gene (Additional file 1: Figure S2).

**Fig. 5:**
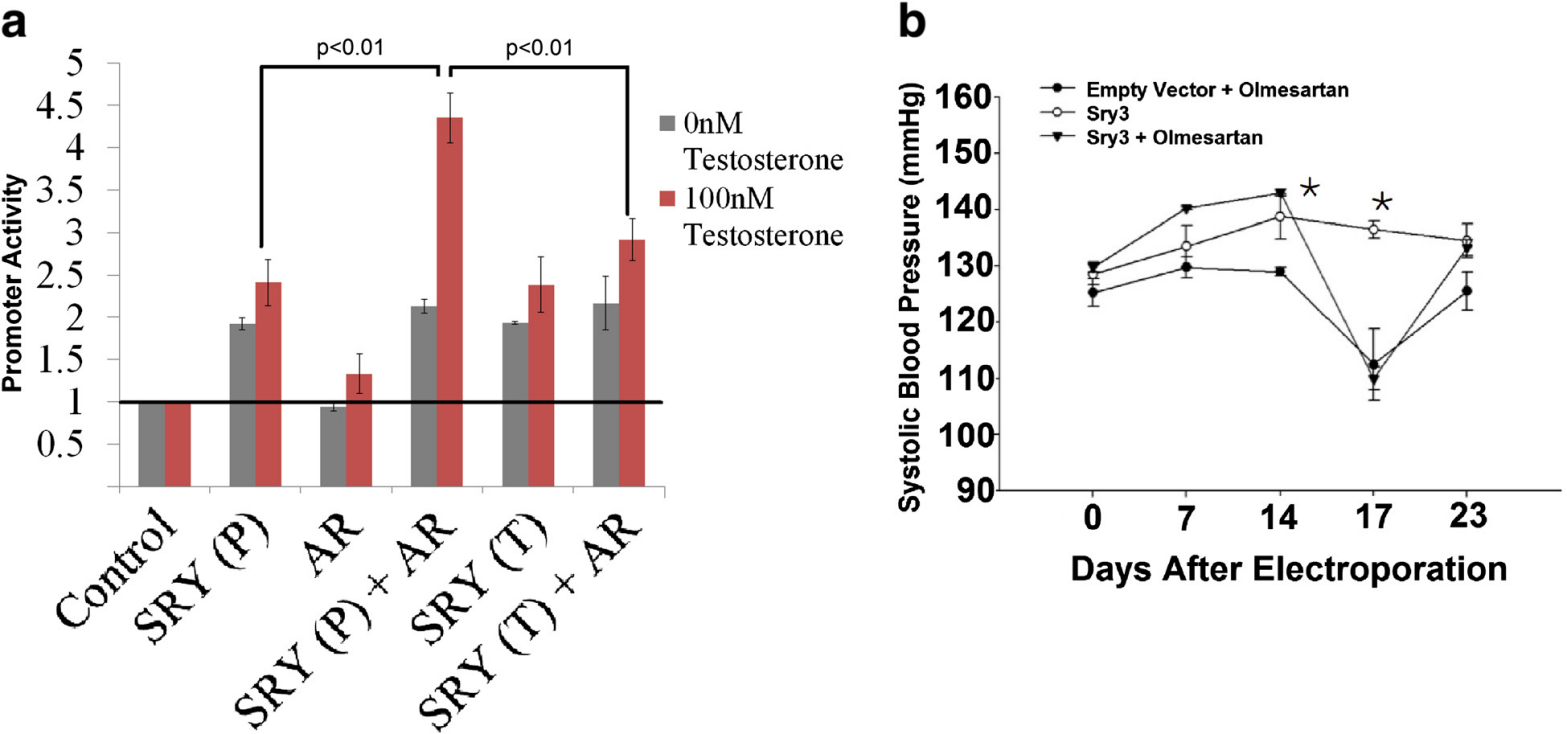
SRY blood pressure regulation through androgen receptor and the renin-angiotensin system. **a** Testosterone-dependent synergistic regulation between SRY and AR is altered by mutations to SRY at the location that separates Sry3 (T) and all other rat and mammalian SRY sequences (P). **b** Delivery of the *Sry3* expression vector (*open circle*) to the kidney of WKY male rats at day 0 significantly increased blood pressure relative to a control (*closed circle*) 14 days after vector electroporation. Olmesartan, a RAS inhibitor, administered to control and half of the Sry3-treated animals (*closed triangle*) at day 14, significantly decreases blood pressure to the same value in both groups. Following removal of olmesartan at day 17, blood pressure increased more rapidly in the *Sry3*-treated group (*closed triangle*). *Error bars* are shown as the SEM of three to four independent animals with *asterisk* representing a *P* < 0.05 for blood pressure between the Sry3 and control vector electroporated animals

Mapping of genes in MSY consomic SHR to WKY crosses (Fig. 1) resolved that the SHR rat has a duplication of an *Sry3* gene relative to other analyzed strains [28]. The cross of the SHR/y consomic animal to the Tfm rat model (Fig. 1) blocks MSY blood pressure elevation, suggesting that there is interplay between the *Sry3* locus and AR signaling. SRY has previously been shown to directly bind AR protein [29]. The SRY3 protein contains an amino acid substitution (proline to threonine) at amino acid 76. Furthermore, we have shown that SRY and AR can synergistically regulate promoters in a testosterone-dependent manner and that a change from a proline to threonine in SRY results in a loss of this synergistic promoter regulation with AR (Fig. 5a). This threonine point mutation has also been shown to increase regulation of the renin-angiotensin system (RAS) components to elevate the pro-hypertensive angiotensin II peptide in vitro [22] and in vivo [23]. *Sry3* copies (*Sry3*, *Sry3A*, *Sry3B*, *Sry3BI*, *Sry3BII*) are expressed in rat (Fig. 4a) suggesting that these *Sry3* genes may have a kidney function through regulation of the RAS. Delivery of a *Sry3* expression vector to the normotensive WKY rat results in a blood pressure elevation that can be blocked with a RAS inhibitor, olmesartan (Fig. 5b). This finding supports *Sry3* gene duplication within SHR causing altered testosterone signaling and regulation of the renin-angiotensin system. In light of preliminary data for the overexpression of human SRY within the rat kidney, this suggests a high probability of SRY involvement in blood pressure regulation and potentially hypertension, with the rat serving to provide mechanistic understanding of SRY within the kidney.

### MSY of other rat strains contributes to phenotypic diversity

**Fig. 6:**
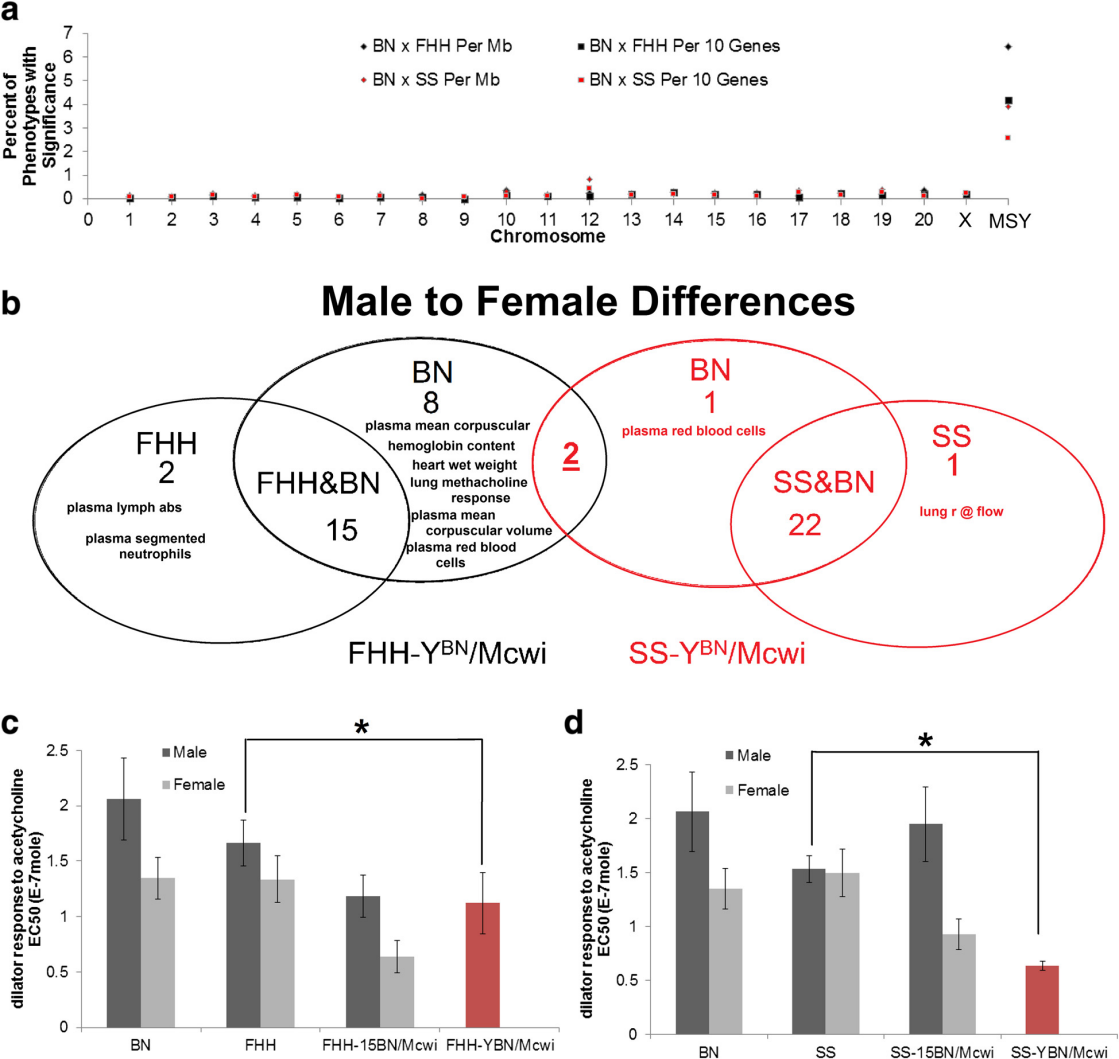
High-throughput phenotyping of two consomic panels in the rat. **a** The percent of significantly altered phenotypes due to crossing each chromosome in two separate consomic panels (BN to FHH in *black* and BN to SS in *red*) shown per megabase (Mb) or per ten genes of the chromosome. **b** Phenotypes identified in **a** to be significantly altered by the two MSY consomic rats (FHH-YBN/Mcwi in *black* and SS-YBN/Mcwi in *red*) were then separated based on if a significant difference between males and females was also seen for one or both strains used to produce the consomic rat. Phenotypes that had a sex difference in one specific strain are listed in each category. Two phenotypes were identified to overlap in the two consomic rats with a BN-specific sex difference, dilator response to acetylcholine EC50 and dilator response to acetylcholine Log EC50. **c**, **d** The dilator response to acetylcholine EC50 for male (*black*) and female (*gray*) BN and FHH (**c**) or SS (**d**) rats showing BN to have the largest sex difference. MSY consomic significantly decreased the response in both FHH (**c**) and SS (**d**) consomic rats (*red*). The chromosome 15 consomic rats (FHH-15BN/Mcwi and SS-15BN/Mcwi) resulted in a greater sex difference for both strains. *Error bars* represent the SEM of independently tested rats and *asterisk* represents an adjusted *P* value <0.05 calculated with Mann-Whitney test followed by a Bonferroni adjustment

Identifying genes in the MSY that contribute to phenotypes such as blood pressure in the rat suggests the need for a broader phenotyping analysis of MSY contribution. As part of two large systematic rat consomic panels created and phenotyped by our PhysGen Program for Genomic Applications [13, 14], data for MSY consomic rats provides the first large-scale phenotyping for MSY contributions. Phenotypes were measured on consomic FHH-Y^BN^/Mcwi (BN MSY introgressed onto FHH genomic background) and SS-Y^BN^/Mcwi (BN MSY introgressed onto SS genomic background). In FHH-Y^BN^/Mcwi and SS-Y^BN^/Mcwi, 131 and 223 phenotypes respectively were measured. When comparing values for FHH-Y^BN^/Mcwi to male FHH rats, 28 phenotypes are significantly different (Additional file 1: Table S5). When comparing values for SS-Y^BN^/Mcwi to male SS rats, 29 phenotypes are significantly different (Additional file 1: Table S6). As a percent of total phenotypes observed to be significantly different in the consomic relative to parental strain per megabase (Mb) of DNA, MSY has a 36.09 ± 3.84-fold (47.23 ± 6.17-fold in BNxFHH and 24.95 ± 3.05-fold in BNxSS) higher contribution to phenotypes than any other chromosome consomic generated (Fig. 6a). Although chromosome size of the MSY differs between rat strains [12], there has yet been evidence of non-MSY sequence driving the changes in size; thus, the genes present in the sequenced SHR are also likely to drive the phenotypes altered by MSY consomics through either copy number variants or SNVs. This is the first evidence that many broad phenotypes in rat lab strains reflect, at least in part, MSY variation.

Additional analysis of MSY contribution can be observed by comparing male to female phenotypes for a particular strain. From the 28 FHH-Y^BN^/Mcwi and 29 SS-Y^BN^/Mcwi significant phenotypes, the difference between male to female parental animals (BN, FHH, and SS) was calculated for each phenotype (Table 3). Of the traits seen significantly altered in FHH-Y^BN^/Mcwi, 15 phenotypes show a significant sex difference in both FHH and BN, while only 2 phenotypes show FHH sex specific differences and 10 phenotypes show BN sex-specific differences (Fig. 6b and Table 3). Of the phenotypes seen significantly altered in SS-Y^BN^/Mcwi, 22 phenotypes show sex differences in both SS and BN, while 1 phenotype shows SS sex-specific difference and 3 phenotypes show BN sex-specific differences (Fig. 6b and Table 3).

**Table 3:** MSY altered phenotypes with differences in male to female rat strains

Phenotype	Rat treatment	Consomic with significant change	Male to female BN significant	Male to female FHH significant	Male to female SS significant
Plasma lymph abs (E3)	12 % O_2_, 0.4 % salt, male	FHH-YBN/Mcwi	No	Yes	–
Plasma segmented neutrophils (E3)	12 % O_2_, 0.4 % salt, male	FHH-YBN/Mcwi	No	Yes	–
Plasma mean corpuscular hemoglobin content (pg)	12 % O_2_, 0.4 % salt, male	FHH-YBN/Mcwi	Yes	No	–
Plasma mean corpuscular volume (fL)	12 % O_2_, 0.4 % salt, male	FHH-YBN/Mcwi	Yes	No	–
Plasma red blood cell (E6/μL)	21 % O_2_, 0.4 % salt, male	FHH-YBN/Mcwi	Yes	No	–
Pre-ischemic heart wet weight (g)	12 % O_2_, 0.4 % salt, male	FHH-YBN/Mcwi	Yes	No	–
Pre-ischemic heart wet weight (g)	21 % O_2_, 0.4 % salt, male	FHH-YBN/Mcwi	Yes	No	–
Pre-ischemic left ventricle developed pressure (mmHg)	12 % O_2_, 0.4 % salt, male	FHH-YBN/Mcwi	Yes	No	–
Pre-ischemic left ventricle systolic pressure (mmHg)	12 % O_2_, 0.4 % salt, male	FHH-YBN/Mcwi	Yes	No	–
Methacholine ED50 (mg/kg)	21 % O_2_, 0.4 % salt, male	FHH-YBN/Mcwi	Yes	No	–
Dilator response to acetylcholine EC50 (E-7 mole)	21 % O_2_, 0.4 % salt, male	FHH-YBN/Mcwi	Yes	No	–
Dilator response to acetylcholine Log EC50 (Log molar)	21 % O_2_, 0.4 % salt, male	FHH-YBN/Mcwi	Yes	No	–
Plasma alk phos (U/L)	21 % O_2_, 0.4 % salt, male	FHH-YBN/Mcwi	Yes	Yes	–
Plasma globulin (g/dL)	21 % O_2_, 0.4 % salt, male	FHH-YBN/Mcwi	Yes	Yes	–
Plasma hematocrit (%)	21 % O_2_, 0.4 % salt, male	FHH-YBN/Mcwi	Yes	Yes	–
Plasma hemoglobin (g/dL)	21 % O_2_, 0.4 % salt, male	FHH-YBN/Mcwi	Yes	Yes	–
Plasma phosphorus (mg/dL)	21 % O_2_, 0.4 % salt, male	FHH-YBN/Mcwi	Yes	Yes	–
Plasma total protein (g/dL)	21 % O_2_, 0.4 % salt, male	FHH-YBN/Mcwi	Yes	Yes	–
Plasma white blood cell count (E3/μL)	12 % O_2_, 0.4 % salt, male	FHH-YBN/Mcwi	Yes	Yes	–
Ischemic peak contracture (mmHg)	12 % O_2_, 0.4 % salt, male	FHH-YBN/Mcwi	Yes	Yes	–
Body weight (kg)	12 % O_2_, 0.4 % salt, male	FHH-YBN/Mcwi	Yes	Yes	–
Body weight (kg)	12 % O_2_, 0.4 % salt, male	FHH-YBN/Mcwi	Yes	Yes	–
Body weight (kg)	21 % O_2_, 0.4 % salt, male	FHH-YBN/Mcwi	Yes	Yes	–
Lung dry wt (g)/body wt. (kg) ratio (g/kg)	21 % O_2_, 0.4 % salt, male	FHH-YBN/Mcwi	Yes	Yes	–
Hematocrit (%)	21 % O_2_, 0.4 % salt, male	FHH-YBN/Mcwi	Yes	Yes	–
Rt ventricle/left ventricle weight ratio (*w*/*w* ratio)	12 % O_2_, 0.4 % salt, male	FHH-YBN/Mcwi	Yes	Yes	–
Body weight	21 % O_2_, 0.4 % salt, male	FHH-YBN/Mcwi	Yes	Yes	–
r @flow = 100 ml/min/g (mmHg × min × kg × ml^−1^)	21 % O_2_, 0.4 % salt, male	SS-YBN/Mcwi	No	–	Yes
Plasma red blood cell (E6/μL)	21 % O_2_, 0.4 % salt, male	SS-YBN/Mcwi	Yes	–	No
Dilator response to acetylcholine EC50 (E-7 mole)	21 % O_2_, 0.4 % salt, male	SS-YBN/Mcwi	Yes	–	No
Dilator response to acetylcholine Log EC50 (Log molar)	21 % O_2_, 0.4 % salt, male	SS-YBN/Mcwi	Yes	–	No
Plasma calcium (mg/dL)	21 % O_2_, 0.4 % salt, male	SS-YBN/Mcwi	Yes	–	Yes
Plasma globulin (g/dL)	21 % O_2_, 0.4 % Salt, Male	SS-YBN/Mcwi	Yes	–	Yes
Plasma globulin (g/dL)	12 % O_2_, 0.4 % salt, male	SS-YBN/Mcwi	Yes	–	Yes
Plasma hematocrit (%)	12 % O_2_, 0.4 % salt, male	SS-YBN/Mcwi	Yes	–	Yes
Plasma hemoglobin (g/dL)	21 % O_2_, 0.4 % salt, male	SS-YBN/Mcwi	Yes	–	Yes
Plasma hemoglobin (g/dL)	12 % O_2_, 0.4 % salt, male	SS-YBN/Mcwi	Yes	–	Yes
Plasma mono abs (E3)	21 % O_2_, 0.4 % salt, male	SS-YBN/Mcwi	Yes	–	Yes
Plasma potassium (mmol/L)	21 % O_2_, 0.4 % salt, male	SS-YBN/Mcwi	Yes	–	Yes
Plasma red blood cell (E6/μL)	12 % O_2_, 0.4 % Salt, Male	SS-YBN/Mcwi	Yes	–	Yes
Plasma total protein (g/dL)	12 % O_2_, 0.4 % salt, male	SS-YBN/Mcwi	Yes	–	Yes
Ischemic time to onset of contracture (s)	12 % O_2_, 0.4 % salt, male	SS-YBN/Mcwi	Yes	–	Yes
Ischemic time to peak contracture (s)	12 % O_2_, 0.4 % salt, male	SS-YBN/Mcwi	Yes	–	Yes
Pre-ischemic heart rate (beats/min)	12 % O_2_, 0.4 % salt, male	SS-YBN/Mcwi	Yes	–	Yes
Pre-ischemic heart wet weight (g)	12 % O_2_, 0.4 % salt, male	SS-YBN/Mcwi	Yes	–	Yes
Body weight (kg)	12 % O_2_, 0.4 % salt, male	SS-YBN/Mcwi	Yes	–	Yes
Body weight (kg)	12 % O_2_, 0.4 % salt, male	SS-YBN/Mcwi	Yes	–	Yes
Lung dry wt (g)/body wt. (kg) ratio (g/kg)	12 % O_2_, 0.4 % salt, male	SS-YBN/Mcwi	Yes	–	Yes
Hematocrit (%)	21 % O_2_, 0.4 % salt, male	SS-YBN/Mcwi	Yes	–	Yes
Hematocrit (%)	12 % O_2_, 0.4 % salt, male	SS-YBN/Mcwi	Yes	–	Yes
Body weight (kg)	21 % O_2_, 0.4 % salt, male	SS-YBN/Mcwi	Yes	–	Yes
Rectal temperature after hypercapnia (°C)	21 % O_2_, 0.4 % salt, male	SS-YBN/Mcwi	Yes	–	Yes
% maximum relaxation acetylcholine (%)	21 % O_2_, 0.4 % salt, male	SS-YBN/Mcwi	Yes	–	Yes

There were two traits that overlapped in both MSY consomic rats that have a BN sex-specific difference, dilator response to acetylcholine EC50, and dilator response to acetylcholine Log EC50. With male BN rats having a higher response than female rats, it is surprising that FHH-Y^BN^/Mcwi (Fig. 6c) and SS-Y^BN^/Mcwi (Fig. 6d) significantly decrease the response relative to either FHH or SS. The large sex difference in the BN rat migrates only with chromosome 15 between the two consomic panels, FHH-15^BN^/Mcwi and SS-15^BN^/Mcwi, suggesting chromosome 15 is responsible for BN-specific sex difference in acetylcholine response, but MSY may have evolved to partially attenuate this response in the BN rat (Fig. 6d, e).

## Discussion

Few animal models currently exist to study the contributions of MSY to phenotypes, which could contribute to narrowing down genes that contribute to human disease. Although many new tools are emerging to study human genetic-to-phenotype associations (such as iPS, organoid culturing, CRISPR/Cas9, and patient cell analysis), studying complex multi-tissue phenotypes and the ability to identify causal variants/genes to disease in a large genomic environment of thousands of inherited variants (such as the MSY) seen in the human still requires animal models. This paper set out to begin defining rat MSY genes in several commonly used rat strains to create models to study MSY variant contributions to phenotypes. The SHR MSY consomic rat (Fig. 1) has served as a model organism for studying the contribution of MSY genes, such as the SHR-specific *Sry3* gene [28], in cardiovascular phenotypes. We have shown in this paper that the protein product of *Sry3* can alter androgen receptor synergistic feedback and that delivery into the rat kidney results in pronounced blood pressure elevation that is blocked by a RAS inhibitor. This suggests a molecular mechanism into the repression of blood pressure response seen in the SHR/y cross to the Tfm rat, showing a synergistic response between MSY genetics and hormone signaling. Our identification of a relationship between genetic variation in rat *Sry* and blood pressure through the RAS promises to facilitate further studies of such a potential mechanistic link between human SRY and blood pressure regulation [22, 27].

Previous data for Sry in both rat and mouse suggest that the gene contributes to diverse phenotypes such as brain development [30] and that MSY genes in the mouse contribute to a number of phenotypes [3]; however, a large-scale phenotyping project has not yet been performed for any consomic animal strains before this paper. In this paper, we have shown that MSY per DNA base has a large phenotypic diversity between rat strains, a likely result of being a region within the genome to have uncontrolled mutations in inbred mating due to a lack of genetic crossover to remove de novo variants. This also highlights the importance of maintaining nomenclature on isolated breeding of strains, as MSY continues to diverge in separate mating locations and can result in different phenotypes based on mating location. This concept is supported by work in comparing the SHR/Akr MSY consomic with the SHR/Crl consomic, which lacks the *Sry3* duplication that results in blood pressure elevation [28] and thus lacks a blood pressure association [31]. This paper would also suggest a potential importance of mitochondrial genomics, as many of the inheritance patterns for that region would be under similar evolution as MSY in inbred animals; however, few studies have focused on this to date.

Furthermore, we have shown the expression profile for nine MSY genes found in rat and human. Using whole-genome sequencing reads from male rats, seven copies of *Sry* have been confirmed for the first time in multiple commonly used laboratory rat strains. The only *Sry* copy found ubiquitously expressed, *Sry2*, was inserted into an intron of the antisense strand of the ubiquitously expressed *Kdm5d* gene and also contains mutations damaging to nuclear localization and transcriptional regulation. With initial fragment analysis protocols only amplifying *Sry2* in an orientation-dependent manner, without amplifying *Kdm5d* introns, the high expression of *Sry2* is likely driven by global chromatin state around the *Kdm5d* gene and is not transcripts detected from Kdm5d intron background of RNAseq. This highlights the fact that the location of genomic insertion can drive an expression profile; however, instead of being able to select on the removal of the gene as would be done on autosomes, MSY sequence has likely been selected on to remove functionality of the protein, maintaining future bulk of MSY contrary to previous MSY degradation theories. Other functional *Sry* copies are found expressed in specific tissues including the testis, kidney, lung, and spleen, similar to the expression profile of human *SRY.* This suggests that “undifferentiated” *Sry* gene copy expression data (generated by techniques such as real-time PCR) biases the understanding of specific *Sry* copies, with the use of sequencing-based technologies or our fragment analysis protocol (can separate out *Sry2* from other copies), a more reliable method for *Sry* expression analysis in future rat work.

The technique of identifying male-specific variants in duplicated genes, although utilized here only in rat, could be used in the future to identify additional mammalian species-specific duplications onto MSY. The assembly of MSYs from several mammals has elucidated duplication events such as *CDY* [32], *FLJ36031Y* [33], *ZNF280AY* [34], and *MBTPS2Y* [1]. However, with only a handful of sequenced MSYs in mammals, the extent of such gene duplications throughout mammalian evolution is unknown. The technique shown here is capable of identifying MSY duplications from reads of several male (or male-contaminated female samples) and female whole-genome sequences, a task that has become relatively inexpensive with next-generation sequencing technologies.

Gene duplication has been recognized as a driver of phenotypic changes for human diseases [35]. Studying duplication of autosomal genes onto MSY provides a unique opportunity to understand mechanisms and selective pressure of gene duplication, in addition to assessing the current status of our genomic scaffold. Of seven duplication events detected in our work, only two genes were found as expressed transcripts, with five becoming pseudogenes. Of the two genes that maintain expression, it is shown that *Med14y* was not present in rat strains such as FHL. Duplication of *Ube2q2*, a gene associated with kidney function [36], onto MSY was initially considered a pseudogene in SHR sequence annotation [2]. However, when analyzing the F344 sequence, transcripts were found ubiquitously expressed from MSY (using the male-specific SNVs). Analysis of 197 male RNAseq datasets from the rat identified 35 additional RNAseq datasets to have detectable *Ube2q2Y* transcripts, suggesting strain-specific stratification in expression. The repetitive nature of rat MSY has made it challenging to generate a complete MSY sequence for rat. The analysis of *Sry* and autosomal gene duplications on MSY can serve as markers for completeness of our current assemblies. For example, the SNVs found in *Sry3C* or pseudogenes *Ect2Y_ps*, *Havcr2Y_ps*, *Prrc2cY_ps*, and *Vom2rY_ps*, which have been confirmed in multiple male rat genomes, are not found in the current Rnor 6.0 MSY assembly. These therefore serve as valuable markers in the future for completing a rat MSY sequence.

## Conclusions

Tools to genetically modify rat strains are rapidly increasing in use [37], allowing for the assessment of a single-gene modification in the vast array of genetic landscapes [15] present in rat research. Identification and characterization of rat MSY genes in this paper opens the door for rat MSY gene editing to study sex differences in diseases. Hopefully, this approach can narrow down the large haplotype block of human MSY to specific genes that contribute to disease association and also suggest approaches that can be used for other species (mouse and primates) to study future MSY phenotype contributions. We now have a causal relationship established in the SHR MSY for the *Sry* gene to cardiovascular disease, allowing for focus on human variants in MSY haplotype groups associated with cardiovascular disease to the *SRY* gene regions, while also implicating AR signaling to influence blood pressure control through SRY synergistic transcriptional regulation.

Utilizing a new male/female SNV segregating approach based on whole-genome sequence reads, we have shown a promising new technique in identifying gene duplications onto MSY that may be critical in identifying species-specific duplication events. Two (*Med14Y* and *Ube2q2Y*) functional MSY retroposed genes (out of ten duplication events shared in most rat strains analyzed) are shown in this paper to have strain-specific variation. The strain-specific outcomes of these duplication events and the high mutation rate of MSY in inbred rat populations suggest a major concern, particularly in light of the phenotypic role MSY is shown to have in this paper. Two consomic panels of inbred rats show MSY to contribute 36-fold more per chromosome size to inbred strain phenotypes than any other chromosome in the rat genome. The combination of approaches taken in this paper to analyze rat MSY genes highlights the importance of MSY to phenotype/disease, suggesting inbred models such as rat are ideal to dissect mechanisms of human MSY genes involved in sex differences. Once gene-to-phenotype relationships are established for these animal models, we envisage that the research community might exploit CRISPR/Cas9 modification of human cell lines to investigate relationships between human MSY variants to disease states.

## Additional file

Additional file 1: Tables S1–S6 and Figures S1–S2.
**Table S1.** Sequences used in BLAST analysis. Table S2. Detection of sex in sequenced rat genomes. **Table S3.** Analysis of SNPs for various rat strains. **Table S4.** Analysis of Sry expression from publically available RNAseq datasets. Table S5. Phenotypes with significant difference between the FHH-YBN/Mcwi (BN Y chromosome consomic with FHH autosomes) relative to the FHH strain out of >200 phenotypes tested. Table S6. Phenotypes that showed a significant difference between the SS-YBN/Mcwi (BN Y chromosome consomic with SS autosomes) relative to the SS strain out of >200 phenotypes tested. **Figure S1.** Limd2y_ps on the Y chromosome. **Figure S2.** NonHMGSry protein models and prediction of functionality. (PDF 1180 kb)

## Abbreviations

AR: Androgen receptor
MSY: Male-specific region of the Y chromosome
RAS: Renin-angiotensin system

## References

[1] Cortez D, Marin R, Toledo-Flores D, Froidevaux L, Liechti A, Waters PD (2014). Origins and functional evolution of Y chromosomes across mammals. Nature.

[2] Bellott DW, Hughes JF, Skaletsky H, Brown LG, Pyntikova T, Cho T-J (2014). Mammalian Y chromosomes retain widely expressed dosage-sensitive regulators. Nature.

[3] Prokop JW, Deschepper CF. Chromosome Y genetic variants: impact in animal models and on human disease. Physiol Genomics. 2015:physiolgenomics.00074.2015. 10.1152/physiolgenomics.00074.2015 PMC4629007 26286457

[4] Forsberg LA, Rasi C, Malmqvist N, Davies H, Pasupulati S, Pakalapati G (2014). Mosaic loss of chromosome Y in peripheral blood is associated with shorter survival and higher risk of cancer. Nat Genet.

[5] Ober C, Loisel DA, Gilad Y (2008). Sex-specific genetic architecture of human disease. Nat Rev Genet.

[6] Rossouw JE (2002). Hormones, genetic factors, and gender differences in cardiovascular disease. Cardiovasc Res.

[7] Bloomer LDS, Nelson CP, Eales J, Denniff M, Christofidou P, Debiec R (2013). Male-specific region of the Y chromosome and cardiovascular risk: phylogenetic analysis and gene expression studies. Arterioscler Thromb Vasc Biol.

[8] Charchar FJ, Bloomer LD, Barnes TA, Cowley MJ, Nelson CP, Wang Y (2012). Inheritance of coronary artery disease in men: an analysis of the role of the Y chromosome. Lancet.

[9] Ely DL, Turner ME (1990). Hypertension in the spontaneously hypertensive rat is linked to the Y chromosome. Hypertension.

[10] Ely DL, Daneshvar H, Turner ME, Johnson ML, Salisbury RL (1993). The hypertensive Y chromosome elevates blood pressure in F11 normotensive rats. Hypertension.

[11] Davidson AO, Schork N, Jaques BC, Kelman AW, Sutcliffe RG, Reid JL (1995). Blood pressure in genetically hypertensive rats. Influence of the Y chromosome. Hypertension.

[12] Kren V, Qi N, Krenova D, Zidek V, Sladká M, Jáchymová M (2001). Y-chromosome transfer induces changes in blood pressure and blood lipids in SHR. Hypertension.

[13] Mattson DL, Dwinell MR, Greene AS, Kwitek AE, Roman RJ, Cowley AW (2007). Chromosomal mapping of the genetic basis of hypertension and renal disease in FHH rats. Am J Physiol Renal Physiol.

[14] Mattson DL, Dwinell MR, Greene AS, Kwitek AE, Roman RJ, Jacob HJ (2008). Chromosome substitution reveals the genetic basis of Dahl salt-sensitive hypertension and renal disease. Am J Physiol Renal Physiol.

[15] Atanur SS, Diaz AG, Maratou K, Sarkis A, Rotival M, Game L (2013). Genome sequencing reveals loci under artificial selection that underlie disease phenotypes in the laboratory rat. Cell.

[16] Yu Y, Fuscoe JC, Zhao C, Guo C, Jia M, Qing T (2014). A rat RNA-Seq transcriptomic BodyMap across 11 organs and 4 developmental stages. Nat Commun.

[17] Turner M, Martin C, Martins A, Dunmire J, Farkas J, Ely D (2007). Genomic and expression analysis of multiple *Sry* loci from a single *Rattus norvegicus* Y chromosome. BMC Genet.

[18] Xu D, Zhang Y (2012). *Ab initio* protein structure assembly using continuous structure fragments and optimized knowledge-based force field. Proteins.

[19] Duan Y, Wu C, Chowdhury S, Lee MC, Xiong G, Zhang W (2003). A point-charge force field for molecular mechanics simulations of proteins based on condensed-phase quantum mechanical calculations. J Comput Chem.

[20] Dinkel H, Michael S, Weatheritt RJ, Davey NE, Roey KV, Altenberg B, et al. ELM—the database of eukaryotic linear motifs. Nucleic Acids Res. 2011:gkr1064. 10.1093/nar/gkr1064 PMC3245074 22110040

[21] Prokop JW, Underwood AC, Turner ME, Miller N, Pietrzak D, Scott S (2013). Analysis of Sry duplications on the Rattus norvegicus Y-chromosome. BMC Genomics.

[22] Prokop JW, Watanabe IKM, Turner ME, Underwood AC, Martins AS, Milsted A (2012). From rat to human: regulation of renin-angiotensin system genes by sry. Int J Hypertens.

[23] Ely D, Boehme S, Dunphy G, Hart M, Chiarappa F, Miller B (2011). The Sry3 Y chromosome locus elevates blood pressure and renin-angiotensin system indexes. Gend Med.

[24] Kwitek AE, Jacob HJ, Baker JE, Dwinell MR, Forster HV, Greene AS (2006). BN phenome: detailed characterization of the cardiovascular, renal, and pulmonary systems of the sequenced rat. Physiol Genomics.

[25] Peng H, Talebzadeh-Farrooji M, Osborne MJ, Prokop JW, McDonald PC, Karar J (2014). LIMD2 is a small LIM-only protein overexpressed in metastatic lesions that regulates cell motility and tumor progression by directly binding to and activating the integrin-linked kinase. Cancer Res.

[26] Chang BH, Shimmin LC, Shyue SK, Hewett-Emmett D, Li WH (1994). Weak male-driven molecular evolution in rodents. Proc Natl Acad Sci U S A.

[27] Araujo FC, Milsted A, Watanabe IKM, Puerto HLD, Santos RAS, Lazar J, et al. Similarities and differences of X and Y chromosome homologous genes, SRY and SOX3, in regulating the renin-angiotensin system promoters. Physiol Genomics. 2015:physiolgenomics.00138.2014. 10.1152/physiolgenomics.00138.2014 PMC4421791 25759379

[28] Turner ME, Farkas J, Dunmire J, Ely D, Milsted A (2008). Which Sry locus is the hypertensive Y chromosome locus?. Hypertension.

[29] Yuan X, Lu ML, Li T, Balk SP (2001). SRY interacts with and negatively regulates androgen receptor transcriptional activity. J Biol Chem.

[30] Turner ME, Ely D, Prokop J, Milsted A (2011). Sry, more than testis determination?. Am J Physiol Regul Integr Comp Physiol.

[31] Vincent M, Kaiser MA, Orea V, Lodwick D, Samani NJ (1994). Hypertension in the spontaneously hypertensive rat and the sex chromosomes. Hypertension.

[32] Lahn BT, Page DC (1999). Retroposition of autosomal mRNA yielded testis-specific gene family on human Y chromosome. Nat Genet.

[33] Murphy WJ, Pearks Wilkerson AJ, Raudsepp T, Agarwala R, Schäffer AA, Stanyon R (2006). Novel gene acquisition on carnivore Y chromosomes. PLoS Genet.

[34] Yang Y, Chang T-C, Yasue H, Bharti AK, Retzel EF, Liu W-S (2011). ZNF280BY and ZNF280AY: autosome derived Y-chromosome gene families in Bovidae. BMC Genomics.

[35] Conrad B, Antonarakis SE (2007). Gene duplication: a drive for phenotypic diversity and cause of human disease. Annu Rev Genomics Hum Genet.

[36] Köttgen A, Pattaro C, Böger CA, Fuchsberger C, Olden M, Glazer NL (2010). New loci associated with kidney function and chronic kidney disease. Nat Genet.

[37] Flister MJ, Prokop JW, Lazar J, Shimoyama M, Dwinell M, Geurts A (2015). 2015 guidelines for establishing genetically modified rat models for cardiovascular research. J Cardiovasc Transl Res.

