# Supplementary material for: The phenotypic impact of the male-specific region of chromosome-Y in inbred mating: the role of genetic variants and gene duplications in multiple inbred rat strains
Source: Biol Sex Differ. 2016 Feb 3;7:10. doi: 10.1186/s13293-016-0064-z (PMC4740989; doi:10.1186/s13293-016-0064-z)
Supplement: Additional file 1: Tables S1–S6 and Figures S1–S2. — Table S1. Sequences used in BLAST analysis. Table S2. Detection of sex in sequenced rat genomes. Table S3. Analysis of SNPs for various rat strains. Table S4. Analysis of Sry expression from publically available RNAseq datasets. Table S5. Phenotypes with significant difference between the FHH-YBN/Mcwi (BN Y chromosome consomic with FHH autosomes) relative to the FHH strain out of >200 phenotypes tested. Table S6. Phenotypes that showed a significant difference between the SS-YBN/Mcwi (BN Y chromosome consomic with SS autosomes) relative to the SS strain out of >200 phenotypes tested. Figure S1. Limd2y_ps on the Y chromosome. Figure S2. NonHMGSry protein models and prediction of functionality. (PDF 1180 kb) [file 13293_2016_64_MOESM1_ESM.pdf]

Table S1 Sequences used in BLAST analysis. Sequences contain SNVs to separate Y-chromosome genes from X- or autosomal genes in BLAST analysis for multiple strains.

Gene	Sequence
DDX3x	GGACATGATGGAGAGAGGGAAGATCGGGCTAGACTTCTGCAAATACCTGGTGTTAGATGAAGCTGACCGGATGT
DDX3Y	GGACATGATGGAAAGAGGAAAGATTGGACTAGATTTCTGCAAATACTTAGTGTTGGATGAAGCTGACAGGATGC
Ect2_2	TAGCTCAGAGGGAAGGAGTCCTCCGAGTAGTGGCAAACTTGCTGTGAGCCGTCTGTCCAG
Ect2_Y	TAGCTCAGAGGGAAaGAGTCCTCgaAGTAGTGGCAAACTTGCTGTGAGCCaTCTGTCCAG
EIF2S3X	TAAGAAGAGGAGTGACCATTAAGCCGACTGTAGATGATGACTGAAGATTCCACGTTAAACAATGCCTTGGGATG
EIF2S3Y	TAAGAAGAGGAGTGACTATCAAGCCAACAATAGATGATGAATGAAATGATACATTTAGGTGAAACCAGAAATG
Havcr2_10	CAGGATTCGGTCTGAGGAAAACATCTACACtATAGAGGAGAACATATATGAAATGGAGAATTCAAAT
Havcr2_Y	CAGGATTCGGTCTGAGGAAAACATCTACACCATAGAGGAGAACATATATGAAATGGAGAATTCAAAT
Kdm5c	ACCAAATTCTTGTGCCCTCTGTGTATGCGCTCACGGCGCCCACGCTTGGAAACCATCCTGGCACTGCTGG
Kdm5d	ACCAAATTCCTGTGCCCACTGTGTATGCGATCAAGGCGTCCACGACTGGAGACCATATTGTCATTGCTGG
Kdm6a	GTTGTGTAACCTTCCACAAGGTAGTCTACAGAATAAAACTAAATTACTTCCTAGTATTGA
Lsm1_16	CATCTGACCAGAGTATCACACTGAAAAGTGATCTTATTCTTTCACAGATGCCAACATGAA
Lsm1_Y	CATCTGACCtGAGTATCACACTGAAAAGTGATCTTATTCTTTCcCAGATGCgAACATGAA
Med14_X	GTTGACGGACTTATTGCCAAGAAAATCGGATGTGGAAAGGAAAATAGAAATTGTACAGTT
Med14_Y	GTTGACGGACTTCTTGCCAAGGAAATCTGATGTGGAAAGGAAAATAGAAATTGTACAGTT
Med14_X_sp	ACACACAGTCTCCAGGAAATCTGCATGCTGCCAGCTCCCCCAGTGGGGCTTTGAGA
Med14_Y_sp	ACACACAGCCTCCAGGAGCCCTTGACTCTAGTTCTCCATACACTCTTATGTCATCA
nonHMGSry	TATGGTGTGGTCCCGCGGAGAGAGGCACAAATGGCCAAATGGCCCAGCAGAATACCCAGC
Not_Sry2	GAGGTCCCGAGGGAGGAGGGATGAATATTTTCTTACACACTTTAAATACTAAAATCTTTG
Not_Sry2_2	GAGCAGTGACAGTTGTCTAGATAGCATGGAGGGCCATGTCAAGCGCCCCATGAATGCATT
Prrc2c_13	CAAAGAGAAATGGAGAGGGCGAGACAGCAAGAGAAAGAGTTAGAACAACAGAGGGAAAAG
Prrc2c_Y	CAAAGAGAAATGGAGAGGGCGAaACAGCAAGAGAAAGAGTTAGAACAACAGAGGGAAAAG
RBMX	GAGACATGAATGGAAAGTCCTTAGATGGAAAAGCCATCAAGGTGGAGCAAGCCACCAAACCATCATTT
RBMY	AAGAAATGAACGGAGTGTCTTTGGATGGAAAAAGAATTAAAGTAGAACAAGCCAGAAGACCATCATCACTT
Sry1	TCAGCATTTCAGATCTTCACTTTCAGTATTCAGCCCTACAGCCTGAGGACATAT
Sry2	GAGGTCCTGAGGGAGAAGGGATGAATATTTTCTTACATACTTTAAATATTAAAATCTTTG
Sry2_2	GAGCAGTGACAGTTGTCTAGATAGCATGGAGGGCCAAGTCAAGCGCCCCATGAATGCATT
Sry3A	ACTGGATACCCACTACAGCAGAAACAGCAGCACCAGGAGCAGCAGCACGTGCACCTGCAG
Sry3B	CAGTCTCTACATTTACAATCCATAGGGGTTTTGGTTGACAATATGTAGGTTTGTGT
Sry3C	ATTTCAGATCTTCACTTTCAGCGTTCAGCCTTCCTTACAGCCTGAGGACATATT
Sry3s	ATATCCAAACTATAAATATCAGACTCATCGAAGGGTTAAAGTGCCACAGAGGAGT
Sry4	TGGCTCTGCTCCTACCTATCCCAACACTCCCCCCTTCTGTAATTTACAGCTTGAGATTCC
Sry4A	TGGCTCTGCTCCTACCTATCCCAACACTCCCCCCTTCTGTTATTTACAGCTTGAGATTCC
Sry4s	AGCGTGAAGTTGCCTCAACAAAAGTGTACAACCTGCTGCAATGGGACAACAACCTACAC
Ssty2_12	TTCTAGCCAAGCCTTCCGTGTACTTCATCAAGTTTGATGGTGACATCCACATCTACATCT
Ssty2_Y	TTCTAGCCAACCTTCCtTGTACTTCATCAAGTTTGATGGcGACATCCgCATCTACATCT
Ube1x	AGCTGCTGTGGCCTCACTCCTACAGTCAGTACAAGTTCCAGAGTTCACCCCCAAGTCTGGTGTCAAGATCCA
Ube1y	TGCTGCTGTGACCACACTCCTGCAGTCTCTGCCAGCCCCCAAGTTTGCTCCCAAGTCTGGCATCAGGATCCA
Ube2q2_8	CTATATTGAGCCTATGGGGTATGAAGATATGCAAAATCCTGTTCGTTTAGAGCCAATAAAAG

Gene	Sequence
**Ube2q2_Y**	CTATATTGAGCCTATGGGGTATGAAGATTTGAAAAATCTTGTTCTTTTAGAGCCAATAAAAG
**Usp9x**	TAATGGGAGATGAACCAGACTTAGATCCTGATATCAATAAGGACTTCTTTGAAAGTAATGTGCTTCAG
**Usp9y**	TAATGGGAGATGAACCTGACTTAGATCCTGATATCAATAAGGAGTTTTTTGAAAGTAATGTGCTTCAG
**UTY**	GTTGTGTAACCTTCCACAAaGTAGTCTACAGAATAAAACTAAATTACTTCCTAGTATTGA
**Vom2r_Y1**	CTGTCCTTCTCGGCCATCACAATTCgAGTcCTAGTCACTTTTGTGAAGTACAAGGATACT
**Vom2r_Y2**	CTGTCCTTCTCaGCCATCACAATTCgAGTtCTAGTCACTTTTGTGAAGTACAAGGATACT
**Vom2r67_14**	CTGTCCTTCTCGGCCATCACAATTCTAGTACTAGTCACTTTTGTGAAGTACAAGGATACT
**Xpr1_13**	TCTGTTTCGAGTATTTACTGCTCCCTTCCATAAGGTAGGCTTTGCTGATTTCTGGCTGGCC
**Xpr1_Y1**	TCTGTTTCcAGTATTTACTGCTCCCTTCCATAAGGTAGGCTTTGCcGATTTCTGGCTGGCC
**Xpr1_Y2**	TCTGTTTCGAGTATTTAGTGCTCCCTTCCATAAGGTAGGCTTTGCcGATTTCTGGCTGGCC
**Xpr1_Y4**	TCTGTTTCGAGTATTTAGTGCTCCCTTCCATAAGGTAGGCTTTGCcGATTTCTGGCTGGCC
**Xpr1_Y5**	TCTGTTTCcAGTATTTCCTGCTCCCTTCCATAAGGTAGGCTTTGCaaATTTCTGcCTGGCC
**ZNFX**	AGGATTCCGAAGACCCTCGGAAAAGAACCAGCACATAATGCGACATCATAAAGAAGTTGGC
**ZNFY**	AGGATTCCGAAGACCCTCtGAAAAGAAtCAGCACATAATGCGgCATCATAAAGAAGTTGGC

Table S2 Detection of sex in sequenced rat genomes. The genome sra reads from multiple rat strains were searched for Sry containing sequences to validate if the sequencing was performed on male (M) or female (F) DNA.

Strain	Experiment	SRA run	Sex
**WAG**	ERX199127	ERR224467	F
**MNS**	ERX199119	ERR224459	F
**MHS**	ERX199118	ERR224458	F
**LE**	ERX199112	ERR224452	F
**BBDP**	ERX199107	ERR224447	F
**WKY**	ERX199130	ERR224470	M
**WKY**	ERX199128	ERR224468	M
**SS**	ERX199126	ERR224466	M
**SS**	ERX199125	ERR224465	M
**SR**	ERX199124	ERR224464	M
**SHRSP**	ERX199123	ERR224463	M
**SHR**	ERX199122	ERR224462	M
**SBN**	ERX199121	ERR224461	M
**SBH**	ERX199120	ERR224460	M
**LL**	ERX199117	ERR224457	M
**LN**	ERX199116	ERR224456	M
**LH**	ERX199115	ERR224455	M
**LEW**	ERX199114	ERR224454	M
**LEW**	ERX199113	ERR224453	M
**FHL**	ERX199110	ERR224450	M
**FHH**	ERX199109	ERR224449	M
**F344**	ERX199108	ERR224448	M
**ACI**	ERX199106	ERR224446	M

Table S3 Analysis of SNPs for various rat strains**.** Sequence alignment of reads from 7 MCW sequenced male rat genomes (ACI, FHH, FHL, SBH, SBN, SR, SS) to the Rattus norvegicus 6.0 genome build including the Y-chromosome of SHR.

Chr	Lengh (Mb)	ACI Total	ACI Density	FHH Total	FHH Density	FHL Total	FHL Density	SBH Total	SBH Density	SBN Total	SBN Density	SR Total	SR Density	SS Total	SS Density
**1**	282.763	477704	1689.41	490586	1734.97	446788	1580.08	487559	1724.27	422814	1495.29	498803	1764.03	463662	1639.75
**2**	266.435	575481	2159.93	571159	2143.71	527985	1981.66	536694	2014.35	516588	1938.89	553531	2077.55	544368	2043.15
**3**	177.700	299255	1684.05	296257	1667.18	280684	1579.54	278393	1566.65	249361	1403.27	306803	1726.52	270304	1521.13
**4**	184.226	290982	1579.48	286575	1555.56	259337	1407.71	252098	1368.41	240093	1303.25	284926	1546.61	277685	1507.30
**5**	173.707	327171	1883.46	325746	1875.26	318163	1831.60	326436	1879.23	298375	1717.69	329197	1895.13	308072	1773.51
**6**	147.991	238352	1610.58	222751	1505.16	218359	1475.48	255832	1728.70	216123	1460.38	247262	1670.79	244728	1653.66
**7**	145.729	288760	1981.48	266226	1826.85	254305	1745.05	258768	1775.68	249945	1715.13	280740	1926.45	264939	1818.02
**8**	133.308	187005	1402.81	178556	1339.43	167410	1255.82	216911	1627.15	202052	1515.68	200478	1503.87	177538	1331.79
**9**	122.095	212552	1740.87	209960	1719.64	188258	1541.89	204361	1673.78	176619	1446.57	196976	1613.30	177415	1453.09
**10**	112.626	178676	1586.45	153061	1359.01	148392	1317.56	166839	1481.35	175127	1554.94	165658	1470.86	155439	1380.13
**11**	90.464	146881	1623.64	108641	1200.93	105041	1161.14	115664	1278.57	121187	1339.62	142768	1578.18	126498	1398.33
**12**	52.717	113985	2162.22	106284	2016.13	103713	1967.36	107659	2042.22	107615	2041.38	115042	2182.27	109783	2082.51
**13**	114.034	194067	1701.84	175234	1536.68	163664	1435.22	183497	1609.14	185022	1622.52	159232	1396.36	153703	1347.87
**14**	115.493	226086	1957.57	213104	1845.16	200079	1732.38	225445	1952.02	221229	1915.51	227940	1973.62	216981	1878.73
**15**	111.246	174966	1572.78	142206	1278.30	128144	1151.90	150669	1354.37	147561	1326.44	171481	1541.45	163096	1466.08
**16**	90.669	175697	1937.79	130895	1443.66	125726	1386.65	151908	1675.42	149471	1648.54	136210	1502.28	120717	1331.41
**17**	90.844	177511	1954.02	165719	1824.22	154580	1701.60	164469	1810.46	150192	1653.30	174091	1916.38	169021	1860.57
**18**	88.202	142044	1610.44	127016	1440.06	126894	1438.68	129266	1465.57	102124	1157.84	133830	1517.31	125336	1421.01
**19**	62.276	111208	1785.74	112713	1809.91	104269	1674.32	104906	1684.54	97702	1568.87	83425	1339.61	86542	1389.66
**20**	56.206	138357	2461.61	118311	2104.95	114408	2035.51	114712	2040.92	113616	2021.42	125703	2236.47	117021	2082.00
**X**	159.970	146582	916.31	122079	763.14	105553	659.83	143369	896.22	121907	762.06	142125	888.45	120513	753.35
**Y**	***3.310***	**11110**	**3356.03**	**12020**	**3630.92**	**7389**	**2232.02**	**9845**	**2973.91**	**6354**	**1919.37**	**15249**	**4606.31**	**9621**	**2906.24**

Table S4 Analysis of Sry expression from publically available RNAseq datasets. RNAseq sra files as reads per million (RPM) analyzed for *Sry2* vs. Not *Sry2* reads using the two SNV locations of *Sry2*.

SRA File	Sry2	Not Sry2	Tissue	Strain
**SRX196304**	6.54E-02	0.273525533	Lung	BN/SsNHsd
**SRX196295**	3.41E-02	0.072482509	Lung	BN/SsNHsd
**SRX265408**	2.65E-02	0.052916078	Prefrontal cortex_Nicotine	F344
**SRX147909**	2.65E-02	0.052916078	Non-pineal mixed tissue	SD
**SRX386107**	2.74E-02	0.027359997	Kidney	
**SRX196300**	1.43E-01	0.027150197	Colon	BN/SsNHsd
**SRX287183**	4.71E-02	0.023570714	Hippocampus_Saline	F344
**SRX265406**	2.04E-02	0.020446297	Retina	SD
**SRX287243**	2.02E-02	0.020151359	Hippocampus_Nicotine	HIV-1Tg
**SRX287216**	0.00E+00	0.017092279	Striatum_Nicotine	HIV-1Tg
**SRX287133**	1.46E-02	0.014559977	Prefrontal cortex_Saline	F344
**SRX196291**	8.85E-02	0.013277709	Colon	BN/SsNHsd
**SRX287123**	1.31E-02	0.013085856	Prefrontal cortex_Saline	F344
**SRX287202**	1.27E-02	0.012699724	Prefrontal cortex_Saline	HIV-1Tg
**SRX287136**	1.16E-02	0.011562926	Prefrontal cortex_Saline	F344
**SRX265404**	2.33E-01	0	Retina	SD
**SRX386103**	2.00E-01	0	Cerebellum	
**SRX288445**	1.84E-01	0	Cerebellum	SD
**SRX205338**	1.24E-01	0	Left ventricle	SD
**SRX265397**	1.12E-01	0	Pineal gland	SD
**ERX305309**	1.10E-01	0	Olfactory epithelium	BN
**ERX305314**	1.09E-01	0	Vomeronasal organs	BN
**ERX270043**	1.06E-01	0	Left ventricle	SHR/Olalpcv
**SRX205337**	1.04E-01	0	Left ventricle	SD
**SRX277015**	9.79E-02	0	Liver_Aflatoxin	F344
**SRX287185**	9.57E-02	0	Hippocampus_Saline	F344
**SRX276999**	9.55E-02	0	Liver_Control	F344
**ERX093544**	9.41E-02	0	Cardiac	RBM20_(-/+)
**ERX093547**	9.24E-02	0	Cardiac	RBM20_(+/+)
**SRX287203**	9.15E-02	0	Prefrontal cortex_Saline	F344
**SRX386105**	8.56E-02	0	Heart	
**ERX270047**	8.21E-02	0	Left ventricle	BN-Lx/Cub
**SRX220847**	8.11E-02	0	Liver	LEW/Crl
**SRX225353**	8.09E-02	0	Embryonic (E15) lateral ventricular choroid plexus	SD
**ERX270049**	8.05E-02	0	Left ventricle	SHR/Olalpcv
**SRX287154**	8.04E-02	0	Striatum_Saline	F344
**ERX305303**	7.95E-02	0	Olfactory epithelium	BN
**ERX305311**	7.92E-02	0	Olfactory epithelium	BN
**SRX287174**	7.92E-02	0	Hippocampus_Nicotine	F344

SRA File	Sry2	Not Sry2	Tissue	Strain
**SRX149624**	7.91E-02	0	Ppineal gland	SD
**SRX149625**	7.82E-02	0	Non-pineal mixed tissue	SD
**SRX287180**	7.82E-02	0	Hippocampus_Saline	F344
**ERX305312**	7.77E-02	0	Vomeronasal organs	BN
**ERX305304**	7.66E-02	0	Olfactory epithelium	BN
**SRX287229**	7.63E-02	0	Striatum_Saline	HIV-1Tg
**ERX093546**	7.42E-02	0	Cardiac	RBM20_(+/+)
**ERX305308**	7.32E-02	0	Vomeronasal organs	BN
**SRX205339**	6.68E-02	0	Left ventricle	SD
**ERX093543**	6.59E-02	0	Cardiac	RBM20_(-/+)
**SRX276998**	6.51E-02	0	Liver	F344
**SRX287255**	6.36E-02	0	Hippocampus_Saline	HIV-1Tg
**SRX287212**	6.30E-02	0	Striatum_Nicotine	HIV-1Tg
**SRX196299**	6.23E-02	0	Brain	BN/SsNHsd
**SRX277017**	6.20E-02	0	FFPE	
**ERX270046**	6.18E-02	0	Left ventricle	SHR/Olalpcv
**ERX093541**	6.15E-02	0	Cardiac	RBM20_(-/-)
**SRX386101**	5.98E-02	0	Frontal cortex	
**SRX386111**	5.96E-02	0	Testis	
**SRX276997**	5.80E-02	0	Liver	F344
**SRX287161**	5.78E-02	0	Striatum_Saline	F344
**SRX287146**	5.69E-02	0	Striatum_Nicotine	F344
**SRX287141**	5.68E-02	0	Striatum_Nicotine	F344
**SRX287143**	5.63E-02	0	Striatum_Nicotine	F344
**SRX287213**	5.63E-02	0	Striatum_Nicotine	HIV-1Tg
**SRX287153**	5.27E-02	0	Striatum_Saline	F344
**SRX287140**	5.24E-02	0	Striatum_Nicotine	F344
**SRX287116**	5.16E-02	0	Prefrontal cortex_Nicotine	F344
**SRX287215**	5.08E-02	0	Striatum_Nicotine	HIV-1Tg
**SRX287120**	4.99E-02	0	Prefrontal cortex_Nicotine	F344
**SRX277016**	4.98E-02	0	FFPE_Aflatoxin	
**SRX287206**	4.88E-02	0	Prefrontal cortex_Saline	HIV-1Tg
**ERX270050**	4.79E-02	0	Left ventricle	BN-Lx/Cub
**SRX463221**	4.78E-02	0	lLver_biliary_fistula_bile	SD
**SRX287184**	4.75E-02	0	Hippocampus_Saline	F344
**SRX287137**	4.68E-02	0	Prefrontal cortex_Saline	F344
**SRX277032**	4.67E-02	0	FFPE_Aflatoxin	
**SRX212314**	4.65E-02	0	Liver	F345
**ERX305313**	4.63E-02	0	Vomeronasal organs	BN
**SRX196290**	4.62E-02	0	Brain	F344/Cr1
**SRX287259**	4.60E-02	0	Hippocampus_Saline	HIV-1Tg
**SRX287156**	4.56E-02	0	Striatum_Saline	F344

SRA File	Sry2	Not Sry2	Tissue	Strain
**ERX305310**	4.56E-02	0	Olfactory epithelium	BN
**SRX265400**	4.44E-02	0	Pineal gland	SD
**SRX265401**	4.43E-02	0	Retina	SD
**SRX287257**	4.36E-02	0	Hippocampus_Saline	HIV-1Tg
**SRX287245**	4.25E-02	0	Hippocampus_Nicotine	HIV-1Tg
**SRX287256**	4.21E-02	0	Hippocampus_Saline	HIV-1Tg
**SRX212317**	4.21E-02	0	Liver	F347
**SRX212316**	4.05E-02	0	Liver	F346
**SRX287254**	4.01E-02	0	Hippocampus_Saline	HIV-1Tg
**SRX105290**	3.96E-02	0	Striatal neural cells_siSupt4h	ST14A cells
**SRX287240**	3.93E-02	0	Hippocampus_Nicotine	HIV-1Tg
**SRX287214**	3.93E-02	0	Striatum_Nicotine	HIV-1Tg
**SRX287145**	3.92E-02	0	Striatum_Nicotine	F344
**SRX287139**	3.89E-02	0	Prefrontal cortex_Saline	F344
**SRX287181**	3.83E-02	0	Hippocampus_Saline	F344
**SRX463219**	3.77E-02	0	Liver_biliary_fistula_bile_infusion	SD
**SRX220846**	3.74E-02	0	Liver	LEW/Crl
**SRX196301**	3.73E-02	0	Heart	BN/SsNHsd
**SRX196297**	3.70E-02	0	Spleen	F344/Cr1
**SRX287218**	3.70E-02	0	Striatum_Nicotine	HIV-1Tg
**SRX220845**	3.64E-02	0	Liver	LEW/Crl
**SRX220851**	3.59E-02	0	Liver	LEW/Crl
**SRX265407**	3.58E-02	0	Non-pineal mixed tissue	SD
**SRX147908**	3.58E-02	0	Non-pineal mixed tissue	SD
**SRX287220**	3.57E-02	0	Striatum_Nicotine	HIV-1Tg
**SRX196306**	3.56E-02	0	Spleen	BN/SsNHsd
**SRX287221**	3.55E-02	0	Striatum_Nicotine	HIV-1Tg
**SRX287191**	3.54E-02	0	Prefrontal cortex_Nicotine	HIV-1Tg
**SRX287228**	3.52E-02	0	Striatum_Saline	HIV-1Tg
**SRX287125**	3.46E-02	0	Prefrontal cortex_Nicotine	F344
**SRX287188**	3.46E-02	0	Prefrontal cortex_Nicotine	HIV-1Tg
**SRX265403**	3.45E-02	0	Retina	SD
**SRX287209**	3.44E-02	0	Prefrontal cortex_Saline	HIV-1Tg
**SRX287231**	3.44E-02	0	Striatum_Saline	HIV-1Tg
**SRX196302**	3.43E-02	0	Kidney	BN/SsNHsd
**SRX287122**	3.39E-02	0	Prefrontal cortex_Nicotine	F344
**SRX287127**	3.37E-02	0	Prefrontal cortex_Nicotine	f344
**SRX212333**	3.33E-02	0	Liver	F348
**SRX212336**	3.29E-02	0	Liver	F350
**SRX105288**	3.21E-02	0	Striatal neural cells	ST14A cells
**SRX265398**	3.13E-02	0	Pineal gland	SD
**ERX270045**	3.07E-02	0	Left ventricle	SHR/Olalpcv

SRA File	Sry2	Not Sry2	Tissue	Strain
**SRX148698**	2.96E-02	0	Vascular smooth muscle cells, AngII-stimulated	SD
**ERX093540**	2.76E-02	0	Cardiac	RBM20_(-/-)
**SRX287187**	2.66E-02	0	Hippocampus_Saline	F344
**SRX225352**	2.61E-02	0	Lateral ventricular choroid plexus	SD
**SRX205341**	2.60E-02	0	Ventricle	SD
**SRX287201**	2.45E-02	0	Prefrontal cortex_Saline	HIV-1Tg
**SRX287186**	2.41E-02	0	Hippocampus_Saline	F344
**SRX287200**	2.40E-02	0	Prefrontal cortex_Saline	HIV-1Tg
**SRX287217**	2.39E-02	0	Striatum_Nicotine	HIV-1Tg
**SRX220850**	2.34E-02	0	Liver	LEW/Crl
**ERX305307**	2.33E-02	0	Vomeronasal organs	BN
**SRX287258**	2.32E-02	0	Hippocampus_Saline	HIV-1Tg
**SRX287147**	2.31E-02	0	Striatum_Nicotine	F344
**SRX287248**	2.20E-02	0	Hippocampus_Saline	HIV-1Tg
**SRX277001**	2.17E-02	0	FFPE	F344
**SRX287149**	2.15E-02	0	Striatum_Nicotine	F344
**SRX287253**	2.13E-02	0	Hippocampus_Saline	HIV-1Tg
**SRX287175**	2.13E-02	0	Hippocampus_Nicotine	F344
**SRX287163**	2.13E-02	0	Striatum_Saline	F344
**SRX287242**	2.12E-02	0	Hippocampus_Nicotine	HIV-1Tg
**SRX287249**	2.11E-02	0	Hippocampus_Saline	HIV-1Tg
**SRX287152**	2.10E-02	0	Striatum_Saline	F344
**SRX287135**	2.09E-02	0	Prefrontal cortex_Saline	F344
**ERX270044**	2.04E-02	0	Left ventricle	BN-Lx/Cub
**SRX287169**	2.04E-02	0	Hippocampus_Nicotine	F344
**SRX287142**	2.03E-02	0	Striatum_Nicotine	F344
**SRX287222**	2.02E-02	0	Striatum_Nicotine	HIV-1Tg
**SRX287158**	2.00E-02	0	Striatum_Saline	F344
**SRX287196**	2.00E-02	0	Prefrontal cortex_Nicotine	HIV-1Tg
**SRX287165**	1.98E-02	0	Hippocampus_Nicotine	F344
**SRX287237**	1.98E-02	0	Hippocampus_Nicotine	HIV-1Tg
**SRX287226**	1.95E-02	0	Striatum_Saline	HIV-1Tg
**SRX287168**	1.95E-02	0	Hippocampus_Nicotine	F344
**SRX287130**	1.94E-02	0	Prefrontal cortex_Saline	F344
**SRX287223**	1.94E-02	0	Striatum_Nicotine	HIV-1Tg
**SRX287171**	1.94E-02	0	Hippocampus_Nicotine	F344
**SRX287182**	1.93E-02	0	Hippocampus_Saline	F344
**SRX287207**	1.93E-02	0	Prefrontal cortex_Saline	HIV-1Tg
**SRX287159**	1.91E-02	0	Striatum_Saline	F344
**SRX287241**	1.86E-02	0	Hippocampus_Nicotine	HIV-1Tg
**SRX287160**	1.86E-02	0	Striatum_Saline	F344
**SRX205343**	1.85E-02	0	Left ventricle	SD

SRA File	Sry2	Not Sry2	Tissue	Strain
**SRX287118**	1.84E-02	0	Prefrontal cortex_Nicotine	F344
**SRX287219**	1.83E-02	0	Striatum_Nicotine	HIV-1Tg
**SRX287173**	1.83E-02	0	Hippocampus_Nicotine	F344
**SRX287126**	1.81E-02	0	Prefrontal cortex_Nicotine	F344
**SRX287192**	1.80E-02	0	Prefrontal cortex_Nicotine	HIV-1Tg
**SRX287129**	1.78E-02	0	Prefrontal cortex_Saline	F344
**SRX287194**	1.68E-02	0	Prefrontal cortex_Nicotine	HIV-1Tg
**SRX287190**	1.68E-02	0	Prefrontal cortex_Nicotine	HIV-1Tg
**SRX287117**	1.66E-02	0	Prefrontal cortex_Nicotine	F344
**SRX287189**	1.66E-02	0	Prefrontal cortex_Nicotine	HIV-1Tg
**SRX287232**	1.63E-02	0	Striatum_Saline	HIV-1Tg
**SRX287177**	1.63E-02	0	Hippocampus_Saline	F344
**SRX287211**	1.63E-02	0	Prefrontal cortex_Saline	HIV-1Tg
**SRX287124**	1.61E-02	0	Prefrontal cortex_Nicotine	F344
**SRX287138**	1.61E-02	0	Prefrontal cortex_Saline	F344
**SRX287199**	1.57E-02	0	Prefrontal cortex_Nicotine	HIV-1Tg
**ERX305306**	1.57E-02	0	Vomeronasal organs	BN
**SRX287121**	1.55E-02	0	Prefrontal cortex_Nicotine	F344
**SRX287134**	1.55E-02	0	Prefrontal cortex_Saline	F344
**SRX196305**	1.54E-02	0	Skeletal muscle	BN/SsNHsd
**SRX287208**	1.52E-02	0	Prefrontal cortex_Saline	HIV-1Tg
**SRX212221**	1.51E-02	0	Liver	F344
**SRX220848**	1.22E-02	0	Liver	LEW/Crl
**ERX270048**	1.15E-02	0	Left ventricle	BN-Lx/Cub
**SRX196303**	1.14E-02	0	Liver	BN/SsNHsd
**SRX463214**	1.05E-02	0	Liver_sham_biliary_fistula	SD
**SRX463222**	9.59E-03	0	Liver_biliary_fistula_bile_infusion	SD
**SRX463220**	9.33E-03	0	Liver_biliary_fistula_bile_infusioL	SD
**SRX463215**	9.20E-03	0	Liver_biliary_fistula_no_bile	SD
**SRX148697**	5.63E-03	0	Vascular smooth muscle cells, untreated	SD
**SRX196293**	4.38E-03	0	Kidney	F344/Cr1
**SRX196307**	4.35E-03	0	Testis	BN/SsNHsd

Table S5 Phenotypes with significant difference between the FHH-Y^{BN}/Mcwi (BN Y-chromosome consomic with FHH autosomes) relative to the FHH strain out of >200 phenotypes tested.

Protocol	Phenotype	Rat treatment	Male to Female FHH Significant	Male to Female BN Significant	FHH Strains Mean +/- FHH SEM	FHH-Y^{BN}/Mcwi Mean +/- FHH-Y^{BN}/Mcwi SEM	Adjusted P Value
Biochemistry	**plasma alk phos (U/L)**	21% O_2, 0.4% Salt, Male	Yes	Yes	358.857 +/- 7.465	426.444 +/- 14.559	0.017573331
Biochemistry	**plasma globulin (g/dL)**	21% O_2, 0.4% Salt, Male	Yes	Yes	1.622 +/- .052	1.267 +/- .041	0.010509682
Biochemistry	**plasma hematocrit (%)**	21% O_2, 0.4% Salt, Male	Yes	Yes	42.713 +/- .444	38.589 +/- .914	0.010034748
Biochemistry	**plasma hemoglobin (g/dL)**	21% O_2, 0.4% Salt, Male	Yes	Yes	13.738 +/- .134	12.433 +/- .261	0.005094596
Biochemistry	**plasma lymph abs (E3)**	12% O_2, 0.4% Salt, Male	Yes	No	4.142 +/- .258	5.884 +/- .27	0.008479522
Biochemistry	**plasma mean corpuscular hemoglobin content(pg)**	12% O_2, 0.4% Salt, Male	No	Yes	18.29 +/- .099	19.211 +/- .131	0.000599037
Biochemistry	**plasma mean corpuscular volume (fL)**	12% O_2, 0.4% Salt, Male	No	Yes	57.316 +/- .299	59.944 +/- .279	0.000800964
Biochemistry	**plasma phosphorus (mg/dL)**	21% O_2, 0.4% Salt, Male	Yes	Yes	9.133 +/- .217	10.722 +/- .329	0.045079426
Biochemistry	**plasma red blood cell (E6/uL)**	21% O_2, 0.4% Salt, Male	No	Yes	7.969 +/- .085	7.204 +/- .161	0.015330947
Biochemistry	**plasma segmented neutrophils (E3)**	12% O_2, 0.4% Salt, Male	Yes	No	.883 +/- .102	1.376 +/- .103	0.0043043
Biochemistry	**plasma total protein (g/dL)**	21% O_2, 0.4% Salt, Male	Yes	Yes	5.412 +/- .076	4.789 +/- .105	0.006804
Biochemistry	**plasma urea nitrogen (mg/dL)**	21% O_2, 0.4% Salt, Male	No	No	17.041 +/- .68	22.556 +/- 1.292	0.008479522
Biochemistry	**plasma white blood cell count (E3/uL)**	12% O_2, 0.4% Salt, Male	Yes	Yes	5.312 +/- .323	7.5 +/- .318	0.003002806
Cardiac	**ischemic peak contracture (mmHg)**	12% O_2, 0.4% Salt, Male	Yes	Yes	33.833 +/- 1.679	21.909 +/- 2.065	0.033986843
Cardiac	**pre-ischemic heart wet weight (g)**	12% O_2, 0.4% Salt, Male	No	Yes	1.001 +/- .04	.654 +/- .023	0.000613737
Cardiac	**pre-ischemic heart wet weight (g)**	21% O_2, 0.4% Salt, Male	No	Yes	.846 +/- .023	.63 +/- .053	0.012758714
Cardiac	**pre-ischemic left ventricle developed pressure (mmHg)**	12% O_2, 0.4% Salt, Male	No	Yes	117.068 +/- 3.066	88.875 +/- 5.762	0.001949975
Cardiac	**pre-ischemic left ventricle systolic pressure (mmHg)**	12% O_2, 0.4% Salt, Male	No	Yes	122.336 +/- 2.988	94.769 +/- 5.775	0.00279609
Cardiac	**body weight (kg)**	12% O_2, 0.4% Salt, Male	Yes	Yes	.14 +/- .005	.098 +/- .003	0.007367112
Lung	**body weight(kg)**	12% O_2, 0.4% Salt, Male	Yes	Yes	.185 +/- .005	.138 +/- .006	0.000427735
Lung	**body weight(kg)**	21% O_2, 0.4% Salt, Male	Yes	Yes	.208 +/- .004	.133 +/- .005	1.1E-10
Lung	**lung dry wt (g)/body wt.(kg) Ratio (g/kg)**	21% O_2, 0.4% Salt, Male	Yes	Yes	.996 +/- .014	1.151 +/- .031	5.81529E-05
Lung	**hematocrit (%)**	21% O_2, 0.4% Salt, Male	Yes	Yes	43.469 +/- .548	38.589 +/- .914	0.001348169
Lung	**methacholine ED50 (mg/kg)**	21% O_2, 0.4% Salt, Male	No	Yes	3.234 +/- .356	1.129 +/- .194	0.005506726
Lung	**rt ventricle/Left ventricle weight ratio (w/w ratio)**	12% O_2, 0.4% Salt, Male	Yes	Yes	.432 +/- .016	.544 +/- .022	0.011906599
Vascular	**dilator response to acetycholine EC50 (E-7mole)**	21% O_2, 0.4% Salt, Male	No	Yes	1.665 +/- .209	1.122 +/- .277	0.012669327
Vascular	**dilator response to acetycholine Log EC50 (Log molar)**	21% O_2, 0.4% Salt, Male	No	Yes	-6.869 +/- .038	-7.09 +/- .073	0.012342305
Vascular	**body weight**	21% O_2, 0.4% Salt, Male	Yes	Yes	.219 +/- .007	.193 +/- .007	0.035170447

Table S6 Phenotypes that showed a significant difference between the SS-Y^{BN}/Mcwi (BN Y-chromosome consomic with SS autosomes) relative to the SS strain out of >200 phenotypes tested.

Protocol	Phenotype	Rat treatment	Male to Female SS Significant	Male to Female BN Significant	SS Strains Mean +/- SS SEM	SS-Y^{BN}/Mcwi Mean +/- SS-Y^{BN}/Mcwi SEM	Adjusted P Value	Shared with FHH-Y^{BN}/Mcwi
Biochemistry	**plasma calcium (mg/dL)**	21% O_2, 0.4% Salt, Male	Yes	Yes	9.408 +/- .064	8.97 +/- .109	0.005018119	
Biochemistry	**plasma globulin (g/dL)**	21% O_2, 0.4% Salt, Male	Yes	Yes	2.172 +/- .032	2.39 +/- .032	0.038230296	**plasma globulin (g/dL)**
Biochemistry	**plasma globulin (g/dL)**	12% O_2, 0.4% Salt, Male	Yes	Yes	2.364 +/- .035	2.625 +/- .026	0.001015626	
Biochemistry	**plasma hematocrit (%)**	12% O_2, 0.4% Salt, Male	Yes	Yes	57.792 +/- .315	61.575 +/- .71	0.000156182	
Biochemistry	**plasma hemoglobin (g/dL)**	21% O_2, 0.4% Salt, Male	Yes	Yes	14.377 +/- .066	14.92 +/- .126	0.007125456	**plasma hemoglobin (g/dL)**
Biochemistry	**plasma hemoglobin (g/dL)**	12% O_2, 0.4% Salt, Male	Yes	Yes	18.633 +/- .104	19.785 +/- .255	0.002506942	
Biochemistry	**plasma mono abs (E3)**	21% O_2, 0.4% Salt, Male	Yes	Yes	.371 +/- .023	.188 +/- .033	0.007533729	
Biochemistry	**plasma potassium (mmol/L)**	21% O_2, 0.4% Salt, Male	Yes	Yes	4.506 +/- .053	5.179 +/- .158	0.000831146	
Biochemistry	**plasma red blood cell (E6/uL)**	12% O_2, 0.4% Salt, Male	Yes	Yes	9.356 +/- .052	10.135 +/- .12	2.69178E-07	
Biochemistry	**plasma red blood cell (E6/uL)**	21% O_2, 0.4% Salt, Male	No	Yes	7.736 +/- .046	8.088 +/- .082	0.005024675	**plasma red blood cell (E6/uL)**
Biochemistry	**plasma total protein (g/dL)**	12% O_2, 0.4% Salt, Male	Yes	Yes	6.068 +/- .04	6.43 +/- .047	0.000310075	
Cardiac	**ischemic time to onset of contracture (sec)**	12% O_2, 0.4% Salt, Male	Yes	Yes	785.963 +/- 19.44	907.966 +/- 40.119	0.002078471	
Cardiac	**ischemic time to peak contracture (sec)**	12% O_2, 0.4% Salt, Male	Yes	Yes	1043.324 +/- 13.268	1174.897 +/- 31.955	0.002023871	
Cardiac	**pre-ischemic heart rate (beats/min)**	12% O_2, 0.4% Salt, Male	Yes	Yes	256.306 +/- 4.646	235.698 +/- 6.255	0.026914713	
Cardiac	**pre-ischemic heart wet weight (g)**	12% O_2, 0.4% Salt, Male	Yes	Yes	1.129 +/- .022	.966 +/- .033	0.002872363	**pre-ischemic heart wet weight (g)**
Cardiac	**body weight (kg)**	12% O_2, 0.4% Salt, Male	Yes	Yes	.199 +/- .004	.168 +/- .005	0.000248443	**body weight (kg)**
Lung	**body weight(kg)**	12% O_2, 0.4% Salt, Male	Yes	Yes	.242 +/- .003	.263 +/- .004	0.002901272	**body weight(kg)**
Lung	**lung dry wt (g)/body wt.(kg) Ratio (g/kg)**	12% O_2, 0.4% Salt, Male	Yes	Yes	1.221 +/- .012	1.095 +/- .011	8.08148E-07	
Lung	**hematocrit (%)**	21% O_2, 0.4% Salt, Male	Yes	Yes	44.515 +/- .269	45.72 +/- .395	0.031294354	**hematocrit (%)**
Lung	**hematocrit (%)**	12% O_2, 0.4% Salt, Male	Yes	Yes	57.967 +/- .344	61.575 +/- .71	0.000238552	
Lung	**r @flow = 100ml/min/g (mmHg x min x kg x ml-1)**	21% O_2, 0.4% Salt, Male	Yes	No	.117 +/- .004	.139 +/- .005	0.013591651	
Respiratory	**body weight (kg)**	21% O_2, 0.4% Salt, Male	Yes	Yes	.296 +/- .003	.326 +/- .004	0.000138023	
Respiratory	**rectal temperature after hypercapnia**	21% O_2, 0.4% Salt, Male	Yes	Yes	36.701 +/- .547	37.637 +/- .148	0.006246728	

Protocol	Phenotype	Rat treatment	Male to Female SS Significant	Male to Female BN Significant	SS Strains Mean +/- SS SEM	SS-Y^{BN}/Mcwi Mean +/- SS-Y^{BN}/Mcwi SEM	Adjusted P Value	Shared with FHH-YBN/Mcwi
Vascular	**dilator response to acetycholine EC50 (E-7mole)**	21% O_2, 0.4% Salt, Male	Yes	Yes	1.529 +/- .124	.633 +/- .042	0.000297425	**dilator response to acetycholine EC50 (E-7mole)**
Vascular	**dilator response to acetycholine Log EC50 (Log molar)**	21% O_2, 0.4% Salt, Male	Yes	Yes	-6.941 +/- .046	-7.214 +/- .028	0.000127294	**dilator response to acetycholine EC50 (E-7mole)**
Vascular	**% maximum relaxation acetylcholine (%)**	21% O_2, 4.0% Salt, Male	No	No	66.833 +/- 2.39	50.667 +/- 2.847	0.037071041	
Vascular	**% maximum relaxation acetylcholine (%)**	21% O_2, 0.4% Salt, Male	Yes	Yes	57.744 +/- 2.411	39.667 +/- 3.709	0.006915885	
Vascular	**% maximum relaxation of phenylephrine-induced contraction by 0% O2 (%)**	21% O_2, 0.4% Salt, Male	No	No	48.6 +/- 2.098	66.2 +/- 3.662	0.000356476	
Vascular	**% maximum relaxation of phenylephrine-induced contraction by 5% O2 (%)**	21% O_2, 4.0% Salt, Male	No	No	32.929 +/- 2.812	46.056 +/- 2.664	0.005250201	

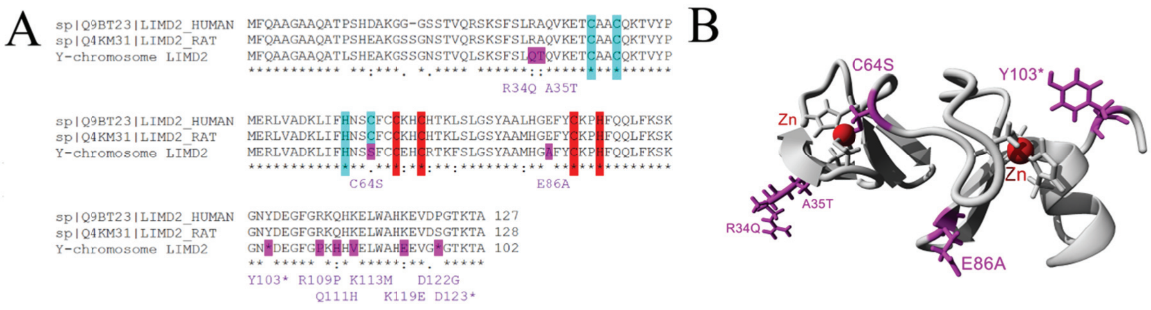

Figure S1 Limd2y_ps on the Y-chromosome.

(A) Sequence alignment of human and rat LIMD2 with the predicted protein product of the rat Y-chromosome gene (LIMD2Y). Amino acids highlighted in cyan and red contribute to the two separate Zn-coordination sites of the LIM domain. Amino acids highlighted in magenta are variants on LIMD2Y. **(B)** Protein structure of human LIMD2 showing the Zn ions (red) and amino acids that are changed in the LIMD2Y predicted protein (magenta).

A

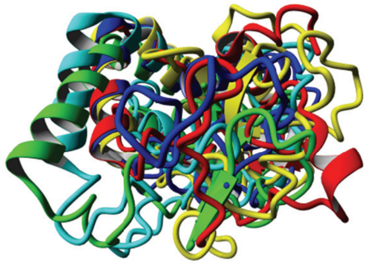

C

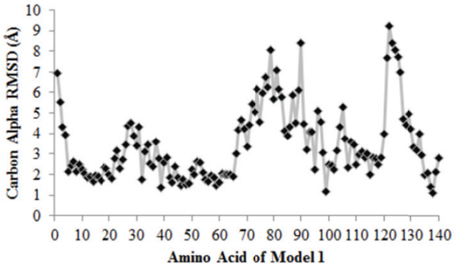

B

	Energy	Z-Score	Wrong Isomers	Cis-peptide bonds	Ca RMSD	Backbone RMSD	RMSF	Net ranking
Model 1	-12582.85	-2.27	0	0	4.017	4.051	2.056	10
Model 2	-11332.58	-2.88	0	0	4.323	4.414	3.007	17
Model 3	-11571.96	-2.57	0	1	3.271	3.368	2.756	12
Model 4	-11202.61	-3.16	0	1	4.391	4.436	4.064	25
Model 5	-11458.55	-2.72	0	0	3.291	3.335	3.394	12

D

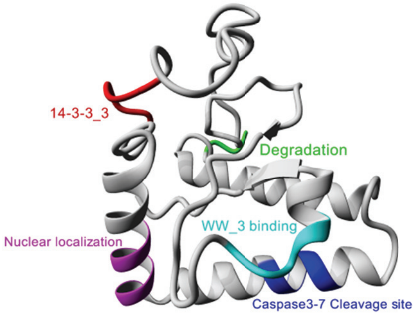

Figure S2 NonHMGSry protein models and prediction of functionality.

(**A)** Structural alignment using the mustang algorithm for the top five models generated by Quark *ab initio* protein modeling. The lack of high structural homology suggests highly divergent structural packing across the models. **(B)** Each of the five models were energy minimized in physiological conditions. The structural Z-score, wrong isomer prediction, and non proline cis-peptide bonds were calculated for each model. Models were then run for 10 nanoseconds of molecular dynamic simulations, calculating the carbon alpha root mean squared deviation (RMSD), backbone RMSD, and the average residue root mean squared fluctuation (RMSF). Model 1 had the lowest ranking and was thus chosen for further analysis. **(C)** Carbon alpha RMSD of model 1 amino acids suggesting structural organization for amino acids 5-66 and 99-118. **(D)** ELM functional motif analysis was performed, mapping the top sites onto the model of the nonHMGSry.
